# Differences in the Abilities to Mechanically Eliminate Activation Energies for Unimolecular and Bimolecular Reactions

**DOI:** 10.1038/srep23059

**Published:** 2016-03-14

**Authors:** Gurpaul S. Kochhar, Nicholas J. Mosey

**Affiliations:** 1 Department of Chemistry, Queen’s University, 90 Bader Lane, Kingston, ON, K7L 3N6, Canada

## Abstract

Mechanochemistry, i.e. the application of forces, *F*, at the molecular level, has attracted significant interest as a means of controlling chemical reactions. The present study uses quantum chemical calculations to explore the abilities to mechanically eliminate activation energies, Δ*E*^‡^, for unimolecular and bimolecular reactions. The results demonstrate that Δ*E*^‡^ can be eliminated for unimolecular reactions by applying sufficiently large *F* along directions that move the reactant and/or transition state (TS) structures parallel to the zero-*F* reaction coordinate, *S*_0_. In contrast, eliminating Δ*E*^‡^ for bimolecular reactions requires the reactant to undergo a force-induced shift parallel to *S*_0_ irrespective of changes in the TS. Meeting this requirement depends upon the coupling between *F* and *S*_0_ in the reactant. The insights regarding the differences in eliminating Δ*E*^‡^ for unimolecular and bimolecular reactions, and the requirements for eliminating Δ*E*^‡^, may be useful in practical efforts to control reactions mechanochemically.

Each step in a chemical reaction involves the progression of a chemical system along a reaction coordinate, *S*, that connects two minima on the potential energy surface (PES). These minima correspond to the reactants and products for that elementary reaction step, while the highest energy structure along *S* is the transition state (TS). The difference in the energies of the TS and reactants is the activation energy, Δ*E*^‡^, for the elementary reaction step and the system must acquire enough energy to overcome Δ*E*^‡^ for the elementary reaction step to occur. Δ*E*^‡^ plays a central role in reaction kinetics. In particular, the rate of an elementary reaction step increases as Δ*E*^‡^ is decreased and the kinetic products of a reaction depend on the relative Δ*E*^‡^s of the slowest steps along the various pathways the reactants may follow. As a result, controlling Δ*E*^‡^ is important in the context of accelerating reactions and selectively forming products.

Changes in Δ*E*^‡^ can be interpreted in terms of the Hammond postulate^1^,^2^, which states that Δ*E*^‡^ decreases as the similarity of the reactant and TS structures increases. Efforts to increase the structural similarity of the reactants and TS without otherwise changing the reaction mechanism involve altering the structures of the reacting species and/or the environment in which the reaction occurs. These changes are achieved through means such as functionalization, changing the solvent in which the reaction is performed, or using catalysts^3^,^4^,^5^,^6^,^7^,^8^,^9^. Recently, advances in experimental techniques for subjecting molecules to tensile forces, *F*, have made it possible to alter reactant and TS structures through mechanical manipulation - an approach termed mechanochemistry^10^,^11^,^12^,^13^,^14^,^15^,^16^,^17^. Mechanochemical methods have been used to lower thermal energy barriers and selectively guide chemical systems along reaction pathways^18^,^19^,^20^,^21^,^22^,^23^,^24^,^25^,^26^,^27^,^28^,^29^,^30^,^31^,^32^. The force-induced reduction in Δ*E*^‡^ in conjunction with a convergence of the reactant and TS structures is evident, for example, from theoretical studies of ring-opening reactions in which the distance separating the reactant and TS along *S* is decreased along with Δ*E*^‡^ as *F* is increased^33^,^34^,^35^. Since *F* is a tunable external parameter, mechanochemistry may offer greater control over the activation of reactions than approaches that alter the molecular structure or environment.

Studies of reactions occurring under mechanochemical conditions have demonstrated that it is possible to eliminate Δ*E*^‡^ by applying sufficiently large *F*^36^,^37^,^38^,^39^. The mathematical definitions of reactants and TSs as minima and first-order saddle points on the PES only permit the elimination of Δ*E*^‡^ through a complete convergence of the reactant and TS structures. This behavior is evident from studies of force-induced ring-opening reactions^33^,^34^,^35^. However, the ability to mechanically render reactions barrierless may apply to unimolecular reactions in a more general sense. Essentially, the reactant and TS structures for unimolecular reactions are separated by a finite distance along *S* in the absence of an applied force, and hence it should be possible in principle to eliminate this separation via mechanical manipulation as long as the system can sustain sufficiently large forces applied along a direction that causes the reactant and TS structures to converge. The ability to eliminate Δ*E*^‡^ in bimolecular reactions occurring under mechanochemical conditions is not as clear. This is particularly true in the case of bimolecular reactions that involve infinitely separated reacting species. In such cases, the infinite distance between the reactants and TS along *S* precludes the convergence of these structures via mechanical manipulation (or any other means). However, many bimolecular reactions include elementary reaction steps that involve progression from a complex to the TS, and may be amenable to the mechanochemical convergence of the reactant and TS structures. In light of these apparent distinctions in the abilities to activate unimolecular and bimolecular reactions, we ask: do differences exist in the abilities to mechanically eliminate Δ*E*^‡^ for unimolecular and bimolecular reactions, and if so, what limitations exist in the ability to render each type of reaction barrierless? The present study answers these questions by using quantum chemical calculations to examine how the thermal energy barriers to a set of representative unimolecular and bimolecular reactions are affected by *F*. The force-induced changes in the thermal energy barriers are interpreted in terms of changes in the separation of the reactant and TS structures along *S*, and shed light on the conditions that must be met to mechanically eliminate Δ*E*^‡^.

## Results

**Figure 1:**
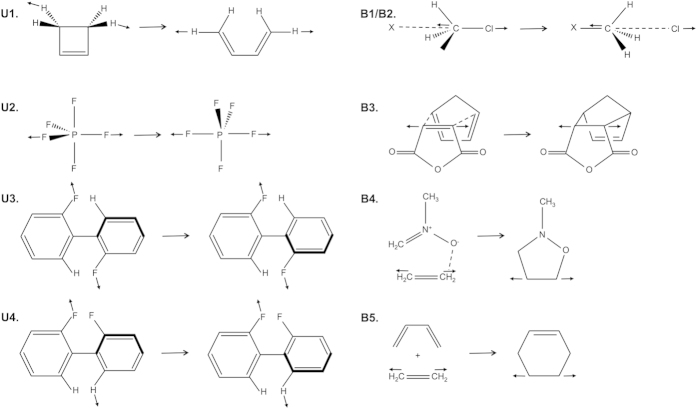
Reactions considered in this study. Reactions **U1** through **U4** correspond to unimolecular reactions. Reactions **B1** through **B4** correspond to bimolecular reactions in which the two reacting components form a complex before reaching the TS. The interactions between these components are indicated with dashed lines. Reaction **B5** corresponds to an addition reaction in which the reacting components are infinitely separated in the reactants. External forces are applied in a tensile manner in all cases, with arrows indicating the atoms used as PPs and the directions of those arrows approximating the direction in which *F* is applied to the PPs.

The force-dependent reactant and TS structures were evaluated for the series of reactions outlined in Fig. 1. Reactions **U1** through **U4** correspond to unimolecular reactions involving bond scission (**U1**), changes in hybridization as the system passes through the TS (**U2**), or changes in conformation without bond dissociation (**U3** and **U4**). Reactions **B1** through **B5** are bimolecular reactions. Reactions **B1** and **B2** correspond to S_N_2 reactions in which the reacting species form a complex. Reactions **B3** through **B5** are cycloaddition reactions, with reactions **B3** and **B4** proceeding via the formation of a reactant complex, and the reacting components being infinitely separated for reaction **B5**. Collectively, these specific reactions are representative of general classes of reactions used extensively in chemical research. For instance, pericyclic reactions (**U1**), substitution reactions (**B1** and **B2**), and cycloadditions (**B3** through **B5**) are used extensively in organic synthesis^40^,^41^,^42^. Furthermore, conformational changes (**U2** through **U4**) play a role in many pharmaceutical and materials applications, with particular connections to chirality^43^,^44^. In addition, pericyclic reactions like **U1** and S_N_2 reactions like **B1** and **B2** have been activated mechanically under experimental conditions^28^,^37^,^45^.

The effects of applied forces on the reactant and TS structures for the reactions in Fig. 1 were evaluated along with the force-dependent changes in Δ*E*^‡^. In all cases, *F* was applied between a pair of atoms, called pulling points (PPs) hereafter, that were selected based on preliminary analyses that indicated applying *F* at these locations had the potential to eliminate Δ*E*^‡^. The magnitude of *F* was increased until TS structures possessing a single normal mode with an imaginary frequency could no longer be located. It is noted that these PPs and magnitudes of *F* may not be accessible in experimental studies of these reactions, but their use was necessary to explore the limits in the ability to mechanically eliminate Δ*E*^‡^. For the same reasons, it was assumed that the reaction mechanism does not change when *F* is applied.

### Unimolecular reactions

**Figure 2:**
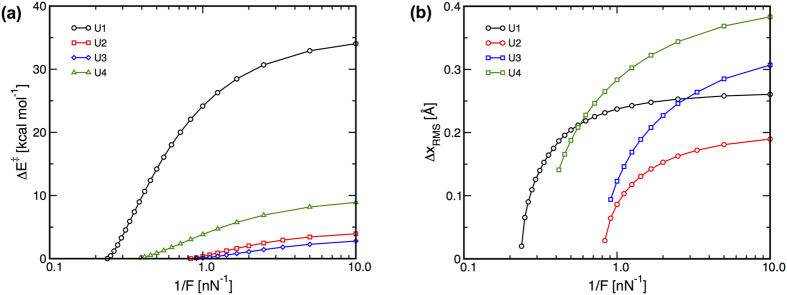
(**a**) Δ*E*^‡^ versus 1/*F* and (**b**) Δ*x*_RMS_ for the unimolecular reactions. The data indicate that Δ*E*^‡^ and Δ*x*_RMS_ tend toward zero at finite *F* for all reactions.

The Δ*E*^‡^s for the unimolecular reactions are plotted versus 1/*F* in Fig. 2a. The data show that the Δ*E*^‡^s approach zero with increasing *F*, with Δ*E*^‡^ reaching values below 0.15 kcal/mol at the highest *F* examined in each case. The differences between the reactant and TS structures were quantified by evaluating the root-mean-squared (RMS) atomic displacements, Δ*x*_RMS_, upon moving from the reactant to the TS at each *F*. The values of Δ*x*_RMS_ (Fig. 2b) show that increasing *F* increases the similarity of the reactant and TS structures, and that Δ*x*_RMS_ should reach zero at finite *F* in all cases if sufficiently large *F* can be supported by the systems. The values of Δ*x*_RMS_ obtained at the highest *F* considered are near zero for reactions **U1** and **U2** (0.020 and 0.029 Å, respectively), which is consistent with the observation that these reactions become effectively barrierless at high *F*. The high-force values of Δ*x*_RMS_ for reactions **U3** and **U4** seem large considering the data in Fig. 2a indicate these reactions become nearly barrierless. This is a result of evaluating the structural similarities using the Cartesian coordinates of the atoms when the structural changes are better described in terms of internal coordinates. Moving from the reactants to the TS for these reactions involves rotation about the central C-C bond of the biaryl. The change in the associated torsion upon moving from the reactants to the TS at the highest *F* considered is relatively small (10.3° and 13.0° for reactions **U3** and **U4**, respectively), yet leads to relatively large changes in the Cartesian positions of the atoms residing far from the rotational axis.

The data in Fig. 2 are consistent with the Hammond postulate, with reductions in Δ*E*^‡^ being associated with decreasing values of Δ*x*_RMS_. The force-induced convergence of the reactant and TS can be achieved through changes in the structure of either one of these species with respect to their locations on the zero-force PES. The displacements of these species parallel (Hammond effects) and perpendicular (anti-Hammond effects) to the zero-force minimum energy path, *S*_0_, were evaluated as follows. First, *S*_0_ was evaluated via an intrinsic reaction coordinate calculation. A stationary point, i.e. a reactant or TS, optimized on the force-modified PES for that reaction was selected and the Δx_RMS_ values based on the difference between the structure of that stationary point and each structure along *S*_0_ were evaluated. The structure, *j*, along *S*_0_ that yielded the lowest value of Δ*x*_RMS_ was deemed most similar to the stationary point. This value of Δ*x*_RMS_, 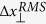, was also used to quantify the amount the stationary point had moved perpendicular to *S*_0_. The value of Δ*x*_RMS_ associated with the difference between structure *j* and the zero-force reactant, 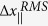, was used to quantify the force-induced deformation of the stationary point parallel to *S*_0_. This procedure was repeated for the stationary points optimized at several *F* for all reactions.

**Figure 3:**
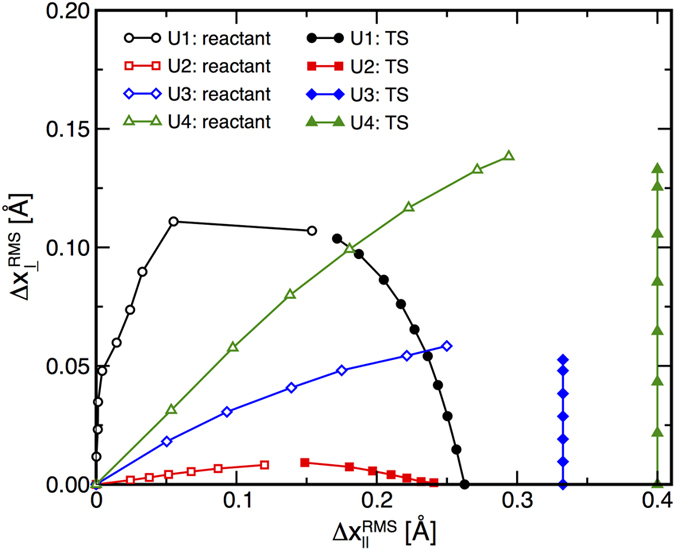
Force-induced deformations of the reactant and TS structures for the unimolecular reactions parallel and perpendicular to the zero-force reaction coordinate, *S*_0_. Open symbols indicate reactant structures and closed symbols designate TS structures optimized at different *F*. Structures obtained at higher *F* lie further from the origins for each curve.

The 
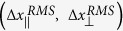
 positions of the stationary points for the unimolecular reactions are given in Fig. 3 and show that the force-induced convergence of the reactant and TS structures is due to a combination of Hammond and anti-Hammond effects. For reactions **U1** and **U2**, *F* shifts the positions of both the reactant and TS structures with respect to *S*_0_. The data for reactions **U3** and **U4** show that the reactants undergo significant force-induced shifts parallel and perpendicular to *S*_0_, while the TS structure does not move parallel to *S*_0_ at all. This behavior can be attributed to *S*_0_ being dominated by rotation about the central C-C bond of the biaryl for these reactions. In the TS, the torsion defined by this bond and the two atoms acting as PPs is 180°, and thus further rotation to move the TS parallel to *S*_0_ would move the PPs closer to one another. Such changes in structure would require work to be performed against *F*, and thus does not occur.

### Bimolecular reactions

The results of quantum chemical calculations of the thermal energy barriers for the bimolecular reactions are reported and examined in what follows. The force-dependent changes in Δ*E*^‡^ and the ability of *F* to increase the similarities of the reactant and TS structures are considered first. The ability of *F* to eliminate Δ*E*^‡^ are then considered in terms of the changes in the deformation and interaction energies of the reacting components as they proceed along the reaction coordinate. The changes illustrate that the reactants must undergo a force-induced shift toward the TS in order for Δ*E*^‡^ to be eliminated mechanically in bimolecular reactions. The ability of the reactants to undertake such a force-induced shift is then considered in terms of the coupling between the direction along with *F* is applied and the zero-force reaction coordinate.

**Figure 4:**
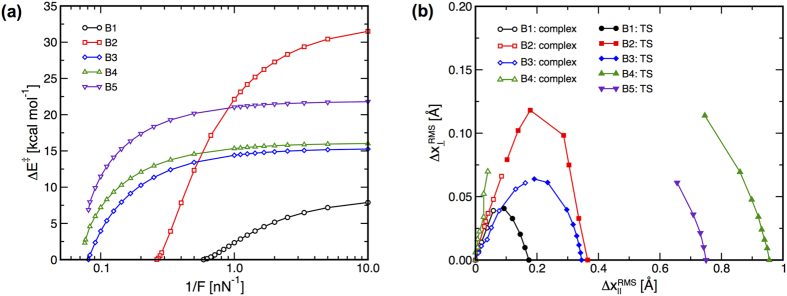
(**a**) Δ*E*^‡^ versus 1/*F* for reactions **B1** through **B5**. (**b**) Force-induced changes of the reactant and TS structures parallel and perpendicular to the zero-force reaction coordinate, *S*_0_. Open symbols indicate reactant structures and closed symbols designate TS structures. The changes in the TS structure for reaction **B5** were obtained by placing the zero-force TS at 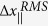 = 0.75 Å. Structures obtained at higher *F* lie further from the origins for each curve.

The Δ*E*^‡^s for the bimolecular reactions are plotted versus 1/*F* in Fig. 4a. For reactions **B1** through **B3**, Δ*E*^‡^ is below 0.25 kcal/mol at the highest *F* that could be supported by the TS. The Δ*E*^‡^s for reactions **B4** and **B5** do not reach zero at the highest values of *F* considered, with the lowest Δ*E*^‡^s achieved for these reactions being 2.3 and 6.9 kcal/mol, respectively. However, the trends in the data suggest that Δ*E*^‡^ should reach zero at finite *F* for both of these reactions. This is reasonable for reaction **B4**, which involves a reactant complex, yet is inconsistent with the infinite separation of the reacting species for reaction **B5**.

The force-induced changes in the reactant and TS structures for the bimolecular reactions relative to *S*_0_ are plotted in Fig. 4b. The data for reactions **B1** through **B3** show that applying *F* causes the reactants and TS to move toward each other along curved paths in the 
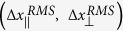
 space. The changes in the reactant and TS structures are relatively symmetric for reactions **B1** and **B3**, whereas the force-induced changes of the TS for reaction **B2** is greater than that for the reactant. The data for reaction **B4** show that *F* induces structural changes in the reactant are nearly perpendicular to the zero-force reactant structure. As a result, any force-induced reduction in the distance between the reactants and TS for this reaction is due primarily to changes in the TS structure. Finally, the TS for reaction **B5** is shifted towards the reactants with increasing *F*; however, the reactants remain infinitely far away.

The force-dependent Δ*E*^‡^s can be represented as the sum of a term corresponding to the change in energy, Δ*E*_def_, associated with structural and electronic changes that occur within each reacting component upon moving from the reactant to TS geometries and another term associated with the change in the interaction energy, Δ*E*_int,_ between the two reacting species as the system moves from the reactants to the TS. The deformation energy is given by:


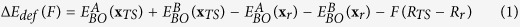


where *A* and *B* designate each of the reacting species, **x**_TS_ and **x**_r_ are the nuclear coordinates of the TS and reactants, respectively, and *R*_TS_ and *R*_r_ are the distances between the PPs in the TS and reactant, respectively. Δ*E*_def_ is necessarily greater than zero in all cases where *A* and *B* are infinitely separated in the reactants, which is true for reaction **B5**. Conversely, the reacting species are present in complexes for reactions **B1** through **B4**, and Δ*E*_def_ is calculated relative to the complex structures for those reactions. In such cases, Δ*E*_def_ is not strictly bound to be greater than zero, but this limit likely still applies because the distance along the reaction coordinate between the complex and completely separated reactants is lower than that between the TS and the completely separated reactants. Once Δ*E*_def_ is calculated, Δ*E*_int_ can be evaluated as 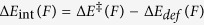. This quantity can take on positive or negative values.

**Figure 5:**
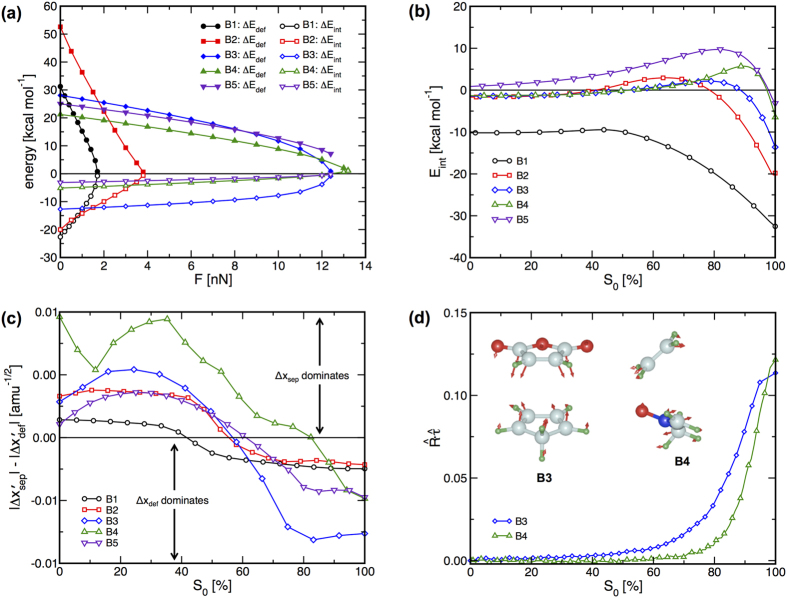
Force-dependent energetic and structural details for reactions B1 through B5. (**a**) Δ*E*_def_ and Δ*E*_int_ versus *F*. (**b**) Interaction energy, *E*_int_, between the reacting components along *S*_0_ relative to the infinitely separated reactants. (**c**) Comparison of rates at which Δ*x*_sep_ and Δ*x*_def_ change along *S*_0_. In each case, *S*_0_ is represented as a percentage of the distance from the reactants (0%) to the TS (100%). (**d**) Projection of *F* onto *S*_0_ for reactions **B3** and **B4**. The reactant complexes for reactions are **B3** and **B4** are shown with arrows indicating the force-induced motions of atoms in those structures, with longer arrows indicating larger motions.

The values of Δ*E*_def_ and Δ*E*_int_ are plotted versus *F* for reactions **B1** through **B5** in Fig. 5a. These data show that Δ*E*_def_ is positive and Δ*E*_int_ is negative at low values of *F* for all reactions. For reactions **B1** through **B3**, increasing *F* lowers Δ*E*_def_ and increases Δ*E*_int_ monotonically such that these quantities each approach zero from opposite sides as *F* approaches the value at which the reactions become barrierless. Similar force-induced changes in Δ*E*_int_ and Δ*E*_def_ occur for reactions **B4** and **B5**; however, in these cases, increasing *F* to the highest values considered causes Δ*E*_int_ to become positive without Δ*E*_def_ reaching zero. The steady reduction in Δ*E*_def_ for these reactions suggests that increasing *F* causes the reacting components to adopt increasingly similar structures in the reactants and TS. In contrast, the adoption of positive values of Δ*E*_int_ indicates that the interactions between these components stabilize the reactants more than the TS at high *F*. Since 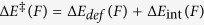, these results suggest that reactions **B4** and **B5** should not become barrierless with increasing *F*.

The results in Fig. 5a highlight that maintaining a negative or zero Δ*E*_int_ is necessary to eliminate Δ*E*^‡^ for bimolecular reactions. The data in Figs 4b and 5a suggest that Δ*E*_int_ remains negative or zero if the reactant experiences a large shift parallel to *S*_0_, e.g. reactions **B1** through **B3**, yet becomes positive if the reactant does not move a significant distance parallel to *S*_0_ irrespective of structural changes in the TS, e.g. reactions **B4** and **B5**. This observation can be understood by examining how the interaction energy, *E*_int_, between the two reacting components changes as the system progresses along *S*_0_ (Fig. 5b). The plots in Fig. 5b show that for all reactions, *E*_int_ increases slowly as the system progresses from the reactants toward the TS before reaching a maximum and then decreasing again as the system moves closer to the TS. In all cases, *E*_int_ is lower at the TS than it is at the reactant, which leads to the negative zero-force values of Δ*E*_int_ for all reactions (Fig. 5a). However, Δ*E*_int_ is a relative quantity that will be affected by the distortions of the reactant and TS structures parallel to *S*_0_. As such, Δ*E*_int_ can become positive if *E*_int_ is greater in the TS than it is in the reactant. This situation can occur if the TS is shifted toward the reactant without a sufficiently large shift of the reactant toward the TS. Interestingly, the shapes of the *E*_int_ curves indicate that positive Δ*E*_int_ should not occur if the TS does not move parallel to *S*_0_. As a whole, these observations lead to the conclusion that the reactant must undergo force-induced change in structure that are directed toward the TS in order to eliminate Δ*E*^‡^ for bimolecular reactions, whereas force-induced changes in the TS structure are not required to eliminate Δ*E*^‡^.

The requirement for the reactant to move toward the TS to eliminate Δ*E*^‡^ is based on the assumption that *E*_int_-*S*_0_ curves possessing the features of those shown in Fig. 5b are common to bimolecular reactions occurring at arbitrary *F*. The origin and generality of these features can be understood by examining the structural changes that occur as the system moves from the reactants to the TS along *S*_0_. This progression can be described in terms of the change in the separation of the reacting species and the deformation of the internal structures of the reacting species. The contributions of each of these types of structural distortions along *S*_0_ were quantified by evaluating the changes in the atomic coordinates of each structure, *j*, along *S*_0_ relative to the reactant complex, *r*, and using these changes to define the total structural change, 
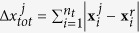
, deformation of the reacting structures, 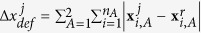, and change in separation 
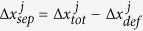
, where *A* indicates the reacting components, *n*_A_ is the number of atoms in component *A*, *n*_t_ is the total number of atoms in the system, 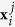 indicates the Cartesian coordinates of the atoms in the structure containing all atoms present in the complex, and 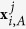 indicates the coordinates of the atoms in the individual reacting components.

The rates at which Δ*x*_sep_ and Δ*x*_def_ change along *S*_0_ are compared in Fig. 5c. In all cases, these data show that changes in Δ*x*_sep_ dominate near the reactants while changes in Δ*x*_def_ dominate closer to the TS. The approach of the two reacting species in the region where Δ*x*_sep_ dominates leads to steric and electrostatic repulsion between valence electrons in these components, which causes *E*_int_ to increase. As the system continues toward the TS and enters the region where Δ*x*_def_ dominates, the initiation of bonding between the reacting species occurs and reduces *E*_int_. This process occurs for the vast majority of bimolecular reactions, and thus the features of the *E*_int_-*S*_0_ curves in Fig. 5b are largely general in nature. The arguments above regarding the changes in Δ*E*_int_ are based on force-induced changes in structure parallel to *S*_0_; however, the data in Fig. 4b indicate that the reactants and TS experience structural changes perpendicular to *S*_0_. Despite these anti-Hammond effects, the progression from a region where Δ*x*_sep_ dominates near the reactants to another where Δ*x*_def_ dominates near the TS occurs for bimolecular reactions at higher *F* if the reactant does not shift significantly toward the TS. The retention of these features at high *F* causes the *E*_int_-*S* curves at higher *F* to resemble those shown in Fig. 5c (see Supplementary Information for examples). In fact, anti-Hammond effects contribute to the steady decrease in Δ*E*_def_ toward zero while Δ*E*_int_ increases that is evident at high *F* for reactions B4 and B5 in Fig. 5a. Such behavior suggests that Δ*E*^‡^ may even begin to increase at higher *F* for these reactions.

The importance of the force-induced distortions of the reactant structure parallel to *S*_0_ in the context of eliminating thermal energy barriers to bimolecular reactions is most apparent in the cases of reactions **B3** and **B4**, which are both pericyclic additions in which the reagents form a complex. Despite this similarity, the differences in the force-induced changes to the reactant structures for these reactions suggest that the nuclear DOFs in these complexes couple to *F* in different ways. This coupling was assessed by calculating the overlap between *F* and *S*_0_ and examining the directions in which the atomic positions in the complexes change due to *F*. The overlap was obtained by evaluating the tangent to *S*_0_ at each point, *i*, along *S*_0_ as:


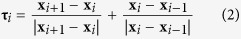


and normalizing as 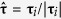 46. The normalized vector, 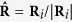, along which *F* is applied was then projected onto 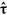 at each point along *S*_0_.

The resulting overlaps are plotted against *S*_0_ in Fig. 5d for reactions **B3** and **B4**. These data show that the overlap between 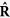 and 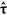 is nearly zero at the reactant structure and increases as the system moves toward the TS. This is consistent with the ability of *F* to move the TS parallel to *S*_0_ for both reactions, but suggests that direct overlap between *F* and *S*_0_ is not responsible for the distortion of the reactant complex parallel to *S*_0_ observed for reaction **B3.** Instead, the structural changes of the reactant complexes are determined by the manner in which *F* couples to other nuclear degrees of freedom (DOFs) in the complexes. This is illustrated by the structures in Fig. 5d, which correspond to the zero-force reactant complexes for these reactions with arrows indicating the magnitudes and directions of the force-induced displacements of each atom. The arrows in each of the reacting components in the complex for reaction **B3** point toward each other, indicating that *F* will shift these components closer to one another, which is consistent with structural changes parallel to *S*_0_. The force-induced changes in the structure show that the coupling between **R** and the other nuclear DOFs causes the C-C distances associated with the formation of products to change at rates of −2.3 × 10^−2 ^Å/nN. Conversely, the arrows on the complex for reaction **B4** do not point toward one another, which would be required for changes in structure parallel to *S*_0_, with the structural changes indicating that the C-O and C-C distances associated with product formation changing at rates of −1.9 × 10^−3^ and −2.2 × 10^−3 ^Å/nN, respectively. Similar analyses show that the reactants and TSs for reactions **B1** and **B2** exhibit analogous features to those illustrated for reaction **B3**. Of course, *F* has no effect on Δ*x*_sep_ for reaction **B5** due to the infinite separation of the reactants.

In a general sense, the ability of *F* to displace the reactant along *S*_0_ in the direction of the TS can assessed by examining the molecular compliance matrix, i.e. the inverse of the molecular Hessian evaluated in terms of internal coordinates^47^,^48^,^49^, and **τ**. Specifically, one can use **τ** to determine which DOFs change significantly as the system follows the reaction coordinate starting at the reactant. Once the relevant DOFs are identified, the elements of the compliance matrix that couple **R** to other nuclear DOFs can be used to quantify the displacement of the system along *S*_0_ when *F* is applied. This approach can also be used to identify PPs that lead to force-induced distortions of the reactant toward the TS.

## Discussion

Collectively, the results of the calculations show that *F* can be used to eliminate Δ*E*^‡^ for elementary reactions by causing the reactant and TS structures to converge through a combination of Hammond and anti-Hammond effects. However, differences in the abilities of *F* to induce the necessary structural convergence exist for unimolecular and bimolecular reactions.

The analysis of the force-induced changes of the reactants and TS structures with respect to *S*_0_ indicates that coupling between *F* and DOFs that cause the reactant structure to shift along S_0_ toward the TS is necessary for the elimination of Δ*E*^‡^ for bimolecular reactions irrespective of the changes in the TS structure. This coupling is absent in the case of bimolecular reactions in which the reactants consist of two infinitely separated molecules. As a result, there will not exist a set of PPs that drives the reactant toward the TS through force-induced reductions in Δ*x*_sep_ (assuming the PPs both reside in one of the reacting molecules), and thus it will not be possible to mechanically eliminate Δ*E*^‡^ for such reactions without changing the mechanism.

In bimolecular reactions in which the reagents form a complex prior to reacting, a finite distance separates the reactants and TS along *S*_0_ and some degree of interaction exists between the two reacting species. As such, these reactions are not necessarily prevented from being rendered barrierless under mechanochemical conditions. In cases where applying *F* to one reagent can drive the entire reactant complex toward the TS due to coupling between *F* and other DOFs that are aligned with *S*_0_, e.g. reactions **B1** through **B3**, the system can retain negative values of Δ*E*_int_ and Δ*E*^‡^ can be eliminated. When the interactions between the components in the complex do not lead to sufficiently coupling between *F* and any DOFs that move the reactant parallel to the reaction coordinate, e.g. reaction **B4**, the system must rely on the changes in the TS structure for the convergence of the reactants and TS to be achieved. As this occurs, the TS samples structures in which Δ*E*_int_ increases without a compensating reduction in Δ*E*_def_, which may even cause Δ*E*^‡^ to increase for certain ranges of increasing *F* and allow Δ*E*^‡^ to reach zero only if the system can sustain sufficiently large *F* to drive the TS all the way to the reactant. However, the lack of coupling between *S* and *F* in the vicinity of the reactant will likely prevent the convergence of the reactant and TS structures as the TS approaches the reactant.

The presence of the reacting species as a single unit in unimolecular reactions renders arguments related to changes in *E*_int_ inapplicable and obviates the need to identify PPs that drive the reactants toward the TS in order to eliminate Δ*E*^‡^. Instead, unimolecular elementary reactions can be rendered barrierless by force-induced changes in either the reactant or TS structures. The presence of strong coupling between the atoms in the molecule undergoing a unimolecular reaction, i.e. coupling sufficiently strong to deem the reactant a single molecule, ensures that it should always be possible in principle to eliminate Δ*E*^‡^ for unimolecular reactions; although, the PPs and *F* required to achieve this may not be experimentally accessible.

Overall, the results of the calculations provide new insights into the ability to mechanically activate reactions by delineating the scenarios in which Δ*E*^‡^ can be mechanically eliminated for common classes of reactions, and relating those abilities to the changes in the reactant and TS structures with respect to *S*_0_. The results are consistent with studies of the unimolecular reactions, which show that the elimination of Δ*E*^‡^ occurs in conjunction with a convergence of the reactant and TS structures^33^,^34^. For bimolecular elementary reactions, the results indicate that the Hammond effects associated with the distortion of the reactant toward the TS are practically necessary to eliminate Δ*E*^‡^ mechanochemically, whereas the changes in the TS structure are less critical in this context. These results can be considered in light of experimental studies showing that reaction barriers can be reduced through force-induced Hammond and anti-Hammond effects^49^,^50^. Such interpretations are based on force-induced changes the separation of the reactant and TS structures along the reaction coordinate, which is sufficient to reduce Δ*E*^‡^. However, the results of the present study indicate that eliminating Δ*E*^‡^ requires the displacement of the reactant along *S*_0_ instead of a consideration of relative structures of the reactants and TS. In addition, models for predicting force-induced changes in Δ*E*^‡^ focus on relative values of properties of the reactant and TS structures^48^,^49^,^51^. Indeed, such models suggest that having a rigid reactant is beneficial for lowering Δ*E*^‡^, whereas the results of the present study suggest that such a scenario would preclude the elimination of Δ*E*^‡^ for some bimolecular reactions. Highlighting the importance of coupling between *F* and DOFs that undergo force-induced motions parallel to *S*_0_ in the reactant may be of practical use in the development of mechanophores and the selection of locations to act as PPs in such molecules. Moreover, illustrating the limitations of the ability to mechanically eliminate Δ*E*^‡^ for bimolecular reactions is of fundamental value.

Of course, the calculations reported above were performed under idealized conditions in which all conceivable sets of PPs could be accessed, constant *F* with magnitudes exceeding those accessible experimentally were applied, and the reaction mechanisms were unaffected by *F*. Despite this idealized scenario, the results of this study are general in nature and apply broadly to the mechanical activation of reactions. Rather deviations from these idealized conditions simply place additional limitations on the ability to mechanically activate reactions. Additionally, the calculations also excluded enthalpic and entropic contributions to reaction kinetics. These factors were not considered in detail through the calculations reported here because of the limited abilities to access enthalpies and free energies via static calculations; however, the mechanical elimination of enthalpic and free energy barriers are subject to the same requirements regarding the convergence of the reactant and TS structures discussed above. In addition, the reactions considered were performed on small model systems that are amenable to computation. Nonetheless, these types of reactions are used extensively in basic and industrial chemical applications^52^,^53^,^54^.

## Methods

The calculations employed the External Force Explicitly Included formalism^51^,^52^ in which *F* is applied along the vector connecting two PPs. Within this formalism, the potential energy of a system on the force-modified PES is:


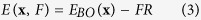


where **x** represents the force-dependent nuclear positions of the atoms in the molecule, *E*_BO_ is the energy of the system on the Born-Oppenheimer PES and *R* is the distance between the PPs. All calculations were performed using Kohn-Sham density functional theory^55^,^56^ with a version of the NWChem 6.1 software package^57^ that we modified to permit single-point energy calculations, geometry optimizations, and frequency calculations on the force-modified PES defined by Eq. (3). The B3LYP exchange-correlation functional^58^,^59^ was used in conjunction with a 6–31G(d,p) basis set for unimolecular reactions and a 6–31++G(d,p) basis set for bimolecular reactions. Calculations with other basis sets and exchange-correlation functionals for selected reactions yielded Δ*E*^‡^ versus *F* relationships that were qualitatively consistent with those reported in the main text. All stationary points were characterized as minima or first-order saddle points on the force-modified PES via harmonic frequency analysis. Values of Δ*E*^‡^ for reaction **B5** were corrected for basis set superposition errors using the counterpoise procedure^60^.

## Additional Information

**How to cite this article**: Kochhar, G. S. and Mosey, N. J. Differences in the Abilities to Mechanically Eliminate Activation Energies for Unimolecular and Bimolecular Reactions. *Sci. Rep.* **6**, 23059; doi: 10.1038/srep23059 (2016).

## Supplementary Material

Supplementary Information
