# Supplementary material for: Differences in the Abilities to Mechanically Eliminate Activation Energies for Unimolecular and Bimolecular Reactions
Source: Sci Rep. 2016 Mar 14;6:23059. doi: 10.1038/srep23059 (PMC4789786; doi:10.1038/srep23059)
Supplement: Supplementary Information [file srep23059-s1.pdf]

Supporting Information for 'Differences in the Abilities to Mechanically Eliminate Activation Energies for Unimolecular and Bimolecular Reactions'

Gurpaul S. Kochhar, and Nicholas J. Mosey

S1. Requirement for convergence of reactant and TS structures to eliminate $\Delta E^{\ddagger}$.

In the main text, it is noted that the mathematical definitions of the reactants and TS only permit the elimination of $\Delta E^{\ddagger}$ in cases where these two species adopt the same structure. The reactant and TS for a reaction step correspond to successive stationary points along the reaction pathway, *S*. The reactant is a local minimum on the potential energy surface (PES), the transition state is a first-order saddle point on this surface and *S* is the minimum energy path that connects these structures. In the event that the reactant and TS are isoenergetic, which would lead to $\Delta E^{\ddagger}$ = 0, the minimum energy path connecting these species would have to be completely flat. Under such conditions, it would not be possible for the curvatures at the reactant and TS structures would be inconsistent with minima or first-order saddle points. Indeed, it would not be possible to designate any structure along the portion of the PES designated *S* in this scenario as a minimum or first-order saddle point. Meanwhile, altering the curvature of the surface along *S* to obtain curvatures consistent with the mathematical definitions of reactant and TS structures would necessarily involve the introduction of additional stationary points, and thus the pair of isoenergetic structures would no longer correspond to the reactant-TS pair for the same reaction step. As a result, $\Delta E^{\ddagger}$ can only reach zero for a given reaction step when the distance between the reactant and TS along *S* is reduced to the point at which these structures converge.

S2. Evaluation of $\Delta x_{\perp}^{RMS}$ and $\Delta x_{\parallel}^{RMS}$.

The *F*-induced movements of the reactant and TS structures relative to the zero-*F* reaction coordinate, S_0, were quantified in the main text through the quantities $\Delta x_{\parallel}^{RMS}$ and $\Delta x_{\perp}^{RMS}$. The manner in which these quantities were calculated is illustrated in what follows using the specific example of the TS for the ring-opening of cyclobutene (reaction **U1**) at *F* = 3000 pN. The structure of this TS, assigned the index *i* hereafter, was used as a reference structure against which the structures, *j*, along S_0 were compared. This structural comparison was quantified by evaluating the root-mean-squared (RMS) differences in the structures of *i* and *j*:

$$\Delta x_{i-j}^{RMS} = \sqrt{\frac{\sum_{k=1}^{3n}\left(x_k^j - x_k^i\right)^2}{3n}} \tag{S1}$$

where the summation runs over all $3n$ Cartesian coordinates for the n atoms in the system, x_k^j is the value of the kth coordinate for the jth structure along S_0 and x_k^i is the value of the kth coordinate for the reference structure, i.

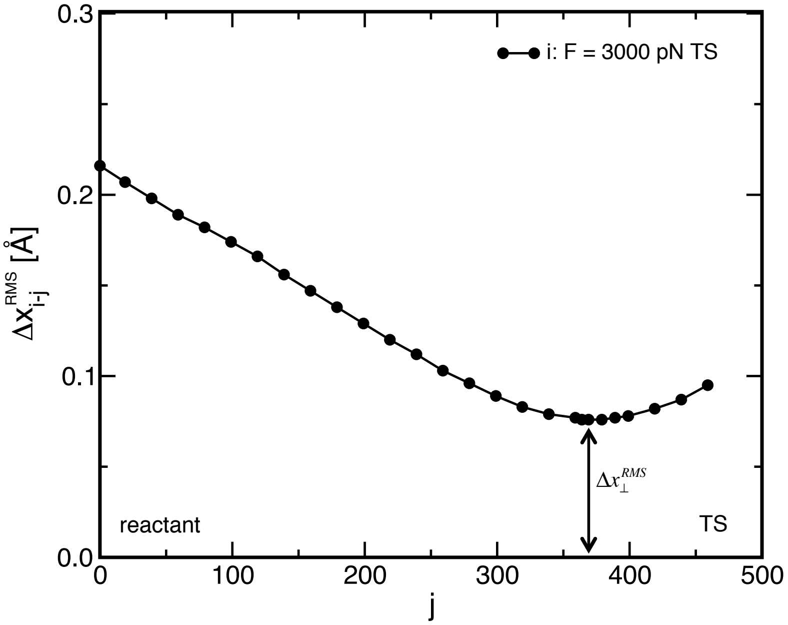

Figure S1. Δx_{i-j}^{RMS} versus j along S_0 for the ring-opening of cyclobutene (reaction **U1**) using the F = 3000 pN TS as the reference structure, i. The index j enumerates the structures along S_0 with the zero-F reactant at j = 0 and the zero-F TS at j = 459. The minimum in Δx_{i-j}^{RMS} is used to identify the structure (j = 369) that is most similar to i. This value of Δx_{i-j}^{RMS} is used to quantify the distance, $\Delta x_{\perp}^{RMS}$, that i resides perpendicular to S_0. The RMS difference between structure j = 369 and the zero-F reactant is then evaluated to determine the position of i parallel to S_0, $\Delta x_{\parallel}^{RMS}$.

A plot of Δx_{RMS}^{i-j} versus j along S_0 is given in Figure S1. The data show that structure i is most similar to structure j = 369 along S_0. The value of Δx_{i-j}^{RMS} associated with i and j = 369 corresponds to $\Delta x_{\perp}^{RMS}$, which quantifies the position of i perpendicular to S_0. The RMS differences between the atomic coordinates of structure j = 369 along S_0 and the zero-F reactant

is used to define the value of $\Delta x_{\|}^{RMS}$, which quantifies the position of i parallel to S_0. In this manner, one can envision reaching structure i by starting at the zero-F reactant, moving an amount $\Delta x_{\|}^{RMS}$ parallel to S_0 to reach structure j = 369 and then moving amount $\Delta x_{\perp}^{RMS}$ perpendicular to S_0 in the direction pointing toward i to reach the TS at F = 3000 pN.

S3. Expectation that ΔE_{def} will be positive for bimolecular reactions with a complex.

In the case of a bimolecular reaction in which the reagents can be treated as infinitely separated species, it is clear that any value of ΔE_{def} calculated relative to the reactant structures will be positive. Essentially, the isolated reactants adopt their minimum energy structures and thus any deformation of those structures will increase their energies. In cases where the reacting species form a complex, one can calculate ΔE_{def} as the sum of the energies associated with deforming these species from their structures in the reactant complex. This was done to evaluate ΔE_{def} for reactions **B1** through **B4**. However, each reacting component does not have its minimum energy configuration, i.e. that it would possess if it was isolated, in the reactant complex. As such, it is possible that the deformation of these structures upon moving from the reactant complex to the TS leads to a reduction in ΔE_{def}. However, given that the reactant complex lies closer to the isolated reactants along the reaction coordinate than the TS does, it is reasonable to assume that the structure of each reactant component is more similar to its isolated structure in the reactant complex than it is in the TS. As a result, it is reasonable to expect ΔE_{def} to be restricted to positive values even in reactions where the reacting components form a complex. This is evident from the data in Figure S2, which show the values of E_{def} relative to the isolated reactants along S_0 for reactions **B1** through **B4**.

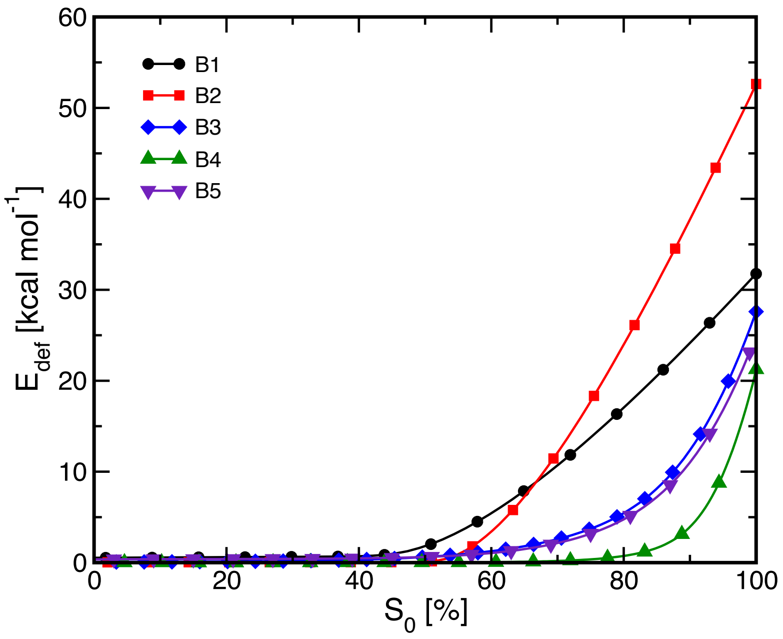

Figure S2. E_{def} versus S_0 for reactions **B1** through **B5**. All energies are calculated relative to the infinitely separated reactants. The results show that $\Delta E_{def} = E_{def}^{TS} - E_{def}^{r}$ is positive in all cases, where the superscripts TS and r indicate the TS and reactant complex structures, respectively. Note that S_0 has been plotted from 0 to 100 %, so the reactant complex resides at 0 % and the TS resides at 100 %.

S4. Changes in E_{int} along the reaction coordinate for bimolecular reactions at different F.

The conclusion that the reactant must move toward the TS in order for ΔE_{int} to remain negative, which allows bimolecular reactions to become barrierless, was based on the notion that the E_{int} changes along the reaction coordinate in the manner shown in Figure 5b of the main text. Those plots were obtained using structures along the zero-F reaction coordinate. Analogous plots at higher F are shown for reactions **B3** and **B4** in Figure S3. In each case, E_{int} was calculated relative to the isolated reactants for structures along the reaction coordinates calculated at different F. The plots were then constructed by plotting the value of E_{int} for each structure at its position parallel to S_0 using the calculated values of $\Delta x_{\parallel}^{RMS}$.

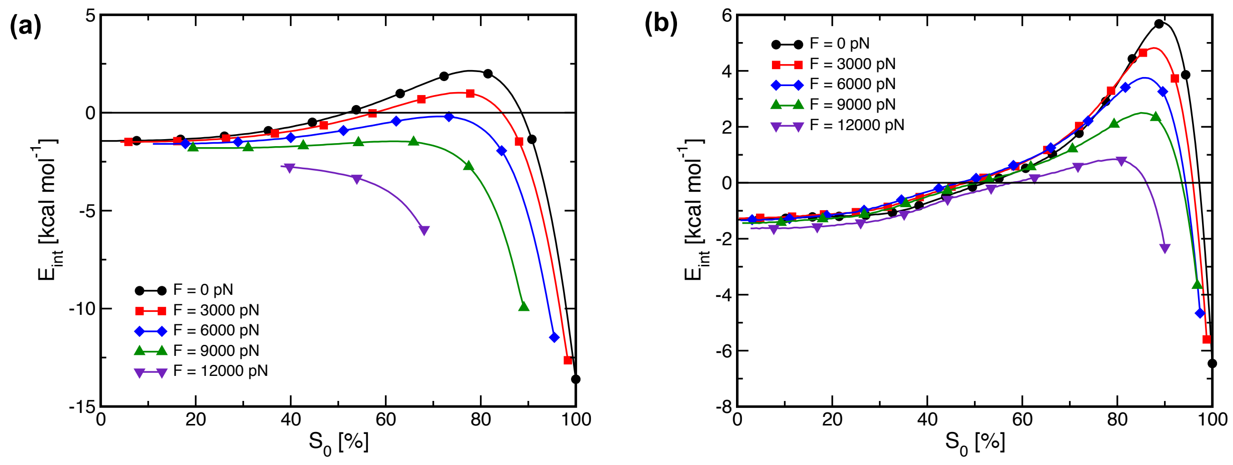

Figure S3. E_{int} versus S_0 for the series of structures along the reaction coordinates for reactions **B3** and **B4** at different values of F. In all cases, E_{int} was calculated relative to the isolated reactants. The positions of the structures along each reaction coordinate are plotted according to their locations parallel to S_0.

The plots for reaction **B3** with F up to 9000 pN have similar shapes, with E_{int} increasing as the system moves from the reactant toward the transition state to reach a maximum value before decreasing again. The maximum value of E_{int} decreases and its position moves closer to the reactant as F increases. In addition, the value of E_{int} at the TS increases with F. The plot at F = 12000 pN does not exhibit a maximum, but rather E_{int} decreases steadily as the system moves from the reactant to the TS. As noted in the main text, the maximum in E_{int} is due to repulsion between the valence electrons of the two reacting species without a sufficiently large stabilization due to bonding or attractive electrostatic interactions between these species, and occurs approximately at the point where the movement along the reaction coordinates changes from being dominated by changes in the separation of the reacting components to structural changes within those components. The E_{int} plot at F = 12000 pN lacks such a maximum because the reactant has undergone a sufficiently large F-induce shift parallel to S_0 to reside at a point where changes in the separation of the reacting species no longer dominate movement along the reaction coordinate. The disappearance of the maximum in E_{int} due to the F-induced movement of the reactant will ensure that ΔE_{int}, i.e. the value of E_{int} in the TS relative to that in the reactant, remains negative at high F.

The plots for reaction **B4** have similar shapes at all *F*. Like reaction **B3**, the maximum value of E_{int} decreases and its position parallel to S_0 shifts toward the reactant with increasing *F*. In addition, the value of E_{int} at the TS increases with *F*. The absence of a significant *F*-induced shift in the position of the reactant causes the value of E_{int} in the reactant to be relatively insensitive to *F*, and does not lead to the elimination of the maximum in E_{int} that occurred for reaction **B4**. Meanwhile, the steady increase in E_{int} for the TS suggests that ΔE_{int} will become positive at some *F*, which was shown in Figure 5a of the main text.

Overall, these data are consistent with the conclusions drawn in the main text. In particular, these data illustrate that the reactant must undergo a shift parallel to S_0 to allow E_{int} to remain negative (or zero), which is necessary for bimolecular reactions to become barrierless. These data also show that anti-Hammond effects affect the values of E_{int}, but these effects are not sufficiently large to obviate the requirement that the reactant moves parallel to S_0 to render a bimolecular reaction barrierless. The similarity between the E_{int} plots obtained at different *F* for reaction **B4** is also consistent with the notion that the *F*-induced movement of the TS alone is not sufficient for the reaction to become barrierless.

S5. Results of test calculations.

The calculations reported in the main text employed the B3LYP exchange-correlation functional[1,2] and 6-31G(d,p) basis set (unimolecular reactions) or 6-31++G(d,p) basis set (bimolecular reactions). The barriers to reactions **U1**, **U2**, **B3**, and **B4** were evaluated with other exchange-correlation functionals including BLYP[2,3] and M06-2X[4] and/or basis sets to assess how the results reported in the main text dependent upon the level of theory used in the calculations. The results of these test calculations are summarized in Figure S4. The results show that all levels of theory yield similar $\Delta E^{\ddagger}$-*F* trends, with differences of up to a few kcal/mol at most in the values of $\Delta E^{\ddagger}$ with changes in level of theory for any given reaction at any given value of *F*. These results indicate that the conclusions reported in this study, which depend upon *F*-induced changes in $\Delta E^{\ddagger}$, are insensitive to the level of theory used in the calculations.

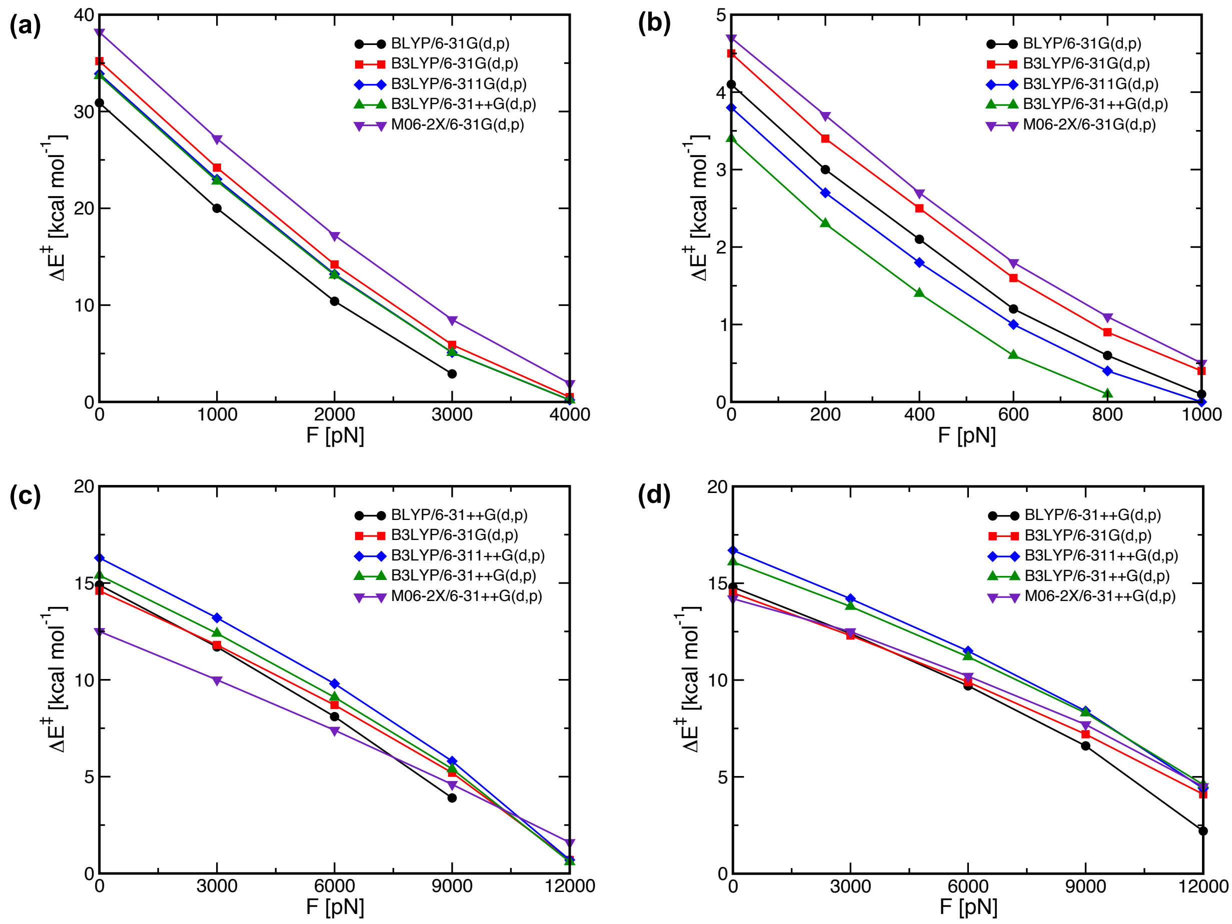

Figure S4. *F*-dependent barriers for reactions (a) **U1**, (b) **U2**, (c) **B3**, and (d) **B4** at different levels of theory.

References.

1. Becke, A. D. Density-functional thermochemistry. III. The role of exact exchange. *J. Chem. Phys.* **98,** 5648–5652 (1993).

2. Lee, C., Yang, W. & Parr, R. G. Development of the Colle-Salvetti correlation-energy formula into a functional of the electron density. *Phys. Rev. B* **37,** 785–789 (1988).

3. Becke, A. D. Density-functional exchange-energy approximation with correct asymptotic behavior. *Phys. Rev. A* **38,** 3098–3100 (1988).

4. Zhao, Y. & Truhlar, D. G. The M06 suite of density functionals for main group thermochemistry, thermochemical kinetics, noncovalent interactions, excited states, and transition elements: two new functionals and systematic testing of four M06-class functionals and 12 other functionals. *Theor. Chem. Acc.* **120,** 215–241 (2008).

S6. Structures and energies.

Ball-and-stick schematics of the reactant and TS structures are shown below of the reactants and transition states for reactions **U1** through **U4** and **B1** through **B5** at *F*=0pN. Black, white, green, orange, blue and red spheres represent carbon, hydrogen, chlorine, phosphorus, nitrogen and oxygen, respectively.

<u>Reaction U1:</u>

Reactant: **TS:**

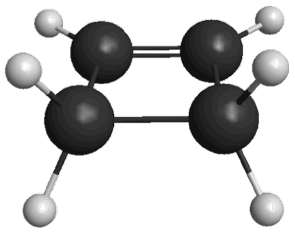

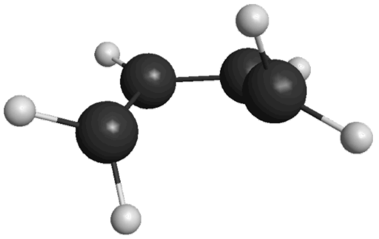

<u>Reaction U2:</u>

Reactant: **TS:**

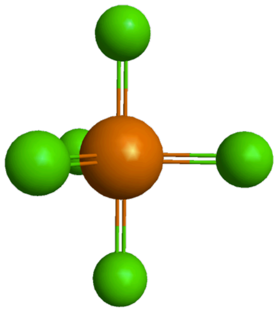

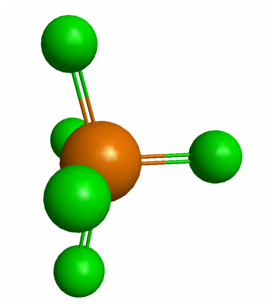

<u>Reaction U3:</u>

Reactant: **TS:**

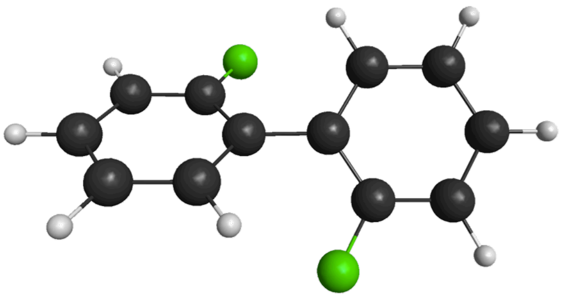

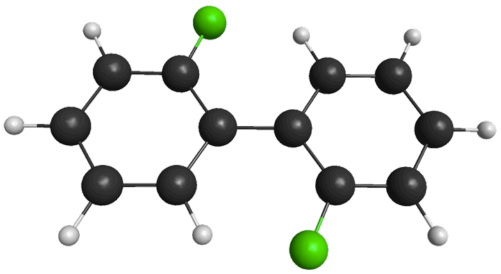

Reaction U4:

Reactant complex:

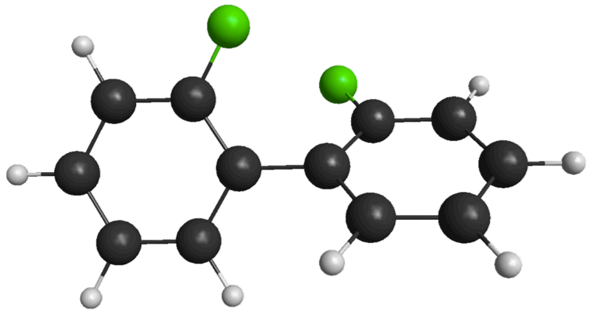

TS:

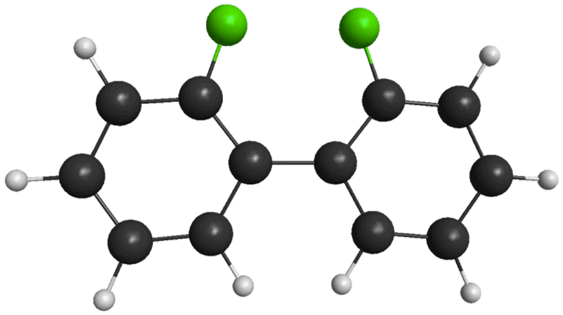

Reaction B1:

Reactant complex:

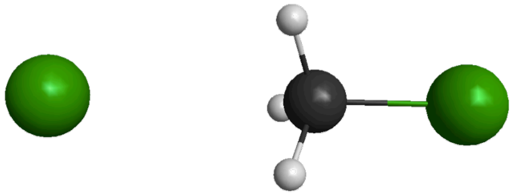

TS:

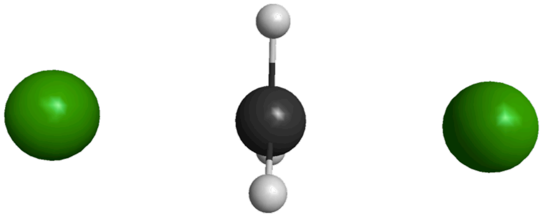

Reaction B2:

Reactant complex:

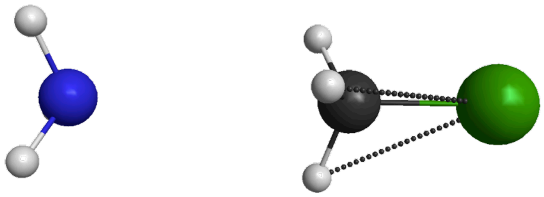

TS:

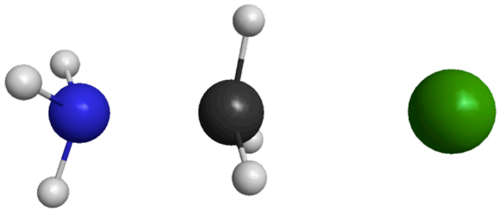

Reaction B3:

Reactant complex:

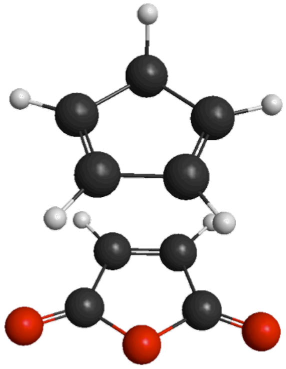

TS:

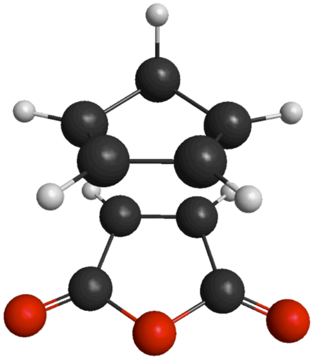

Reaction B4:

Reactant complex:

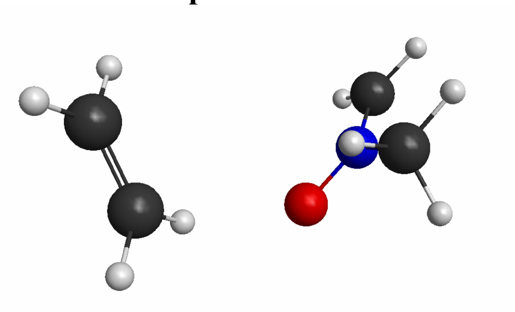

TS:

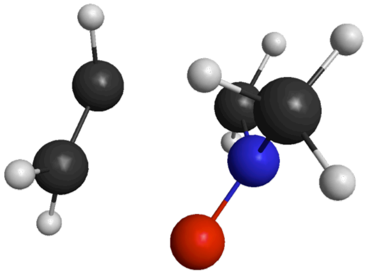

Reaction B5:

1,3-butadiene:

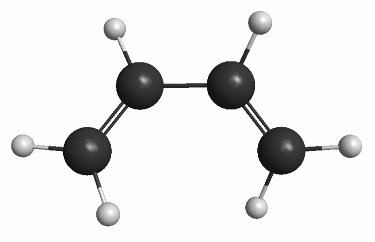

TS:

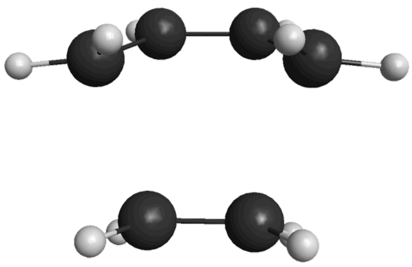

Ethylene:

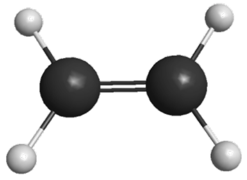

Cartesian coordinates and energies on the force-modified PES are shown below of the reactants and transition states for reactions **U1** through **U4** and **B1** through **B5** at selected values of *F*.

Reaction U1:

Reactants (PPs = atoms 6 and 9):

F = 0 pN
EFEI energy (au): -155.98232414
Coordinates (Å):

H	0.0000	-1.4195	1.6008
C	0.0000	0.6701	0.8145
C	0.0000	-0.6701	0.8145
C	0.0000	0.7862	-0.6994
C	0.0000	-0.7862	-0.6994
H	0.8893	-1.2464	-1.1457
H	-0.8893	-1.2464	-1.1457
H	0.0000	1.4195	1.6008
H	-0.8893	1.2464	-1.1457
H	0.8893	1.2464	-1.1457

F = 1000 pN
EFEI energy (au): -156.05409171
Coordinates (Å):

H	0.0046	-1.4177	1.5991
C	0.0009	0.6695	0.8116
C	-0.0009	-0.6695	0.8116
C	-0.0099	0.7968	-0.7021
C	0.0099	-0.7968	-0.7021
H	0.9068	-1.3153	-1.1086
H	-0.8783	-1.2602	-1.1477
H	-0.0046	1.4177	1.5991
H	-0.9068	1.3153	-1.1085
H	0.8784	1.2602	-1.1477

F = 2000 pN
EFEI energy (au): -156.12894058
Coordinates (Å):

H	0.0196	-1.4147	1.5978
C	-0.0028	0.6689	0.8081
C	0.0028	-0.6689	0.8081
C	-0.0208	0.8112	-0.7039
C	0.0208	-0.8112	-0.7038
H	0.9191	-1.3897	-1.0793
H	-0.8710	-1.2733	-1.1441

H	-0.0196	1.4147	1.5978
H	-0.9192	1.3897	-1.0793
H	0.8710	1.2732	-1.1441

F = 3000 pN
EFEI energy (au): -156.20712858
Coordinates (Å):

H	0.0601	-1.4095	1.5827
C	-0.0181	0.6681	0.7897
C	0.0181	-0.6681	0.7897
C	-0.0337	0.8324	-0.7182
C	0.0337	-0.8324	-0.7182
H	0.9214	-1.4813	-1.0753
H	-0.8724	-1.2841	-1.1398
H	-0.0601	1.4095	1.5827
H	-0.9214	1.4813	-1.0753
H	0.8724	1.2841	-1.1398

F = 4000 pN
EFEI energy (au): -156.28944577
Coordinates (Å):

H	0.1354	-1.3954	1.5737
C	-0.0468	0.6662	0.7729
C	0.0468	-0.6662	0.7729
C	-0.0520	0.8741	-0.7238
C	0.0520	-0.8741	-0.7237
H	0.9152	-1.6125	-1.0652
H	-0.8786	-1.2972	-1.1185
H	-0.1353	1.3954	1.5737
H	-0.9152	1.6126	-1.0653
H	0.8786	1.2972	-1.1185

Transition states (PPs = atoms 6 and 9):

F = 0 pN
EFEI energy (au): -155.92623751
Coordinates (Å):

H	1.3399	1.5411	-0.4008
C	-0.6827	0.7337	0.0843
C	0.6834	0.7331	-0.0843
C	-1.0644	-0.6252	-0.1179
C	1.0639	-0.6262	0.1180
H	1.8783	-1.1026	-0.4370
H	0.8662	-1.0863	1.0780
H	-1.3386	1.5423	0.4008
H	-1.8792	-1.1009	0.4371

H	-0.8672	-1.0856	-1.0780

F = 1000 pN
EFEI energy (au): -156.01559184
Coordinates (Å):

H	1.3474	1.5472	-0.3301
C	-0.6781	0.7294	0.0694
C	0.6788	0.7288	-0.0694
C	-1.0506	-0.6534	-0.1043
C	1.0500	-0.6543	0.1043
H	1.9105	-1.0823	-0.4567
H	0.9471	-1.0804	1.0963
H	-1.3460	1.5484	0.3300
H	-1.9115	-1.0806	0.4567
H	-0.9480	-1.0796	-1.0963

F = 2000 pN
EFEI energy (au): -156.10630552
Coordinates (Å):

H	1.3562	1.5533	-0.2529
C	-0.6745	0.7293	0.0509
C	0.6751	0.7287	-0.0509
C	-1.0336	-0.6779	-0.0943
C	1.0330	-0.6788	0.0943
H	1.9279	-1.0696	-0.4826
H	1.0185	-1.0746	1.1063
H	-1.3548	1.5545	0.2529
H	-1.9289	-1.0679	0.4825
H	-1.0195	-1.0737	-1.1064

F = 3000 pN
EFEI energy (au): -156.19770269
Coordinates (Å):

H	1.3678	1.5801	-0.1644
C	-0.6715	0.7555	0.0263
C	0.6722	0.7549	-0.0263
C	-1.0061	-0.6783	-0.0875
C	1.0056	-0.6791	0.0875
H	1.9248	-1.0429	-0.5180
H	1.0796	-1.0526	1.1083
H	-1.3665	1.5812	0.1644
H	-1.9256	-1.0413	0.5180
H	-1.0805	-1.0517	-1.1083

F = 4000 pN
EFEI energy (au): -156.28872778

Coordinates (Å):

H	1.3849	1.5880	-0.0414
C	-0.6684	0.7719	-0.0130
C	0.6691	0.7713	0.0131
C	-0.9509	-0.6949	-0.0850
C	0.9503	-0.6958	0.0850
H	1.8777	-1.0403	-0.5737
H	1.1274	-1.0630	1.0991
H	-1.3835	1.5893	0.0414
H	-1.8786	-1.0386	0.5737
H	-1.1283	-1.0620	-1.0991

Reaction U2:

Reactants (PPs = atoms 3 and 5):

F = 0 pN
EFEI energy (au): -840.67634337
Coordinates (Å):

P	0.0018	0.0001	-0.0015
F	0.6832	1.2660	0.6942
F	0.1249	-0.8042	1.3404
F	1.1648	-0.0478	-1.0540
F	-1.2842	0.8512	-0.2920
F	-0.6792	-1.2657	-0.6972

F = 300 pN
EFEI energy (au): -840.69521902
Coordinates (Å):

P	-0.0038	0.0000	0.0033
F	0.6637	1.2658	0.7108
F	0.1576	-0.8199	1.3379
F	1.1581	-0.0473	-1.0477
F	-1.2774	0.8660	-0.3245
F	-0.6981	-1.2648	-0.6800

F = 600 pN
EFEI energy (au): -840.71445978
Coordinates (Å):

P	-0.0079	0.0004	0.0072
F	0.6427	1.2660	0.7291
F	0.1975	-0.8382	1.3315
F	1.1523	-0.0472	-1.0426
F	-1.2665	0.8818	-0.3645
F	-0.7181	-1.2628	-0.6608

F = 900 pN
EFEI energy (au): -840.73411099
Coordinates (Å):

P	-0.0123	0.0006	0.0113
F	0.6143	1.2656	0.7532
F	0.2473	-0.8595	1.3210
F	1.1459	-0.0470	-1.0368
F	-1.2503	0.9000	-0.4140
F	-0.7448	-1.2599	-0.6348

F = 1200 pN
EFEI energy (au): -840.75428424
Coordinates (Å):

P	-0.0182	0.0007	0.0165
F	0.5589	1.2628	0.7982
F	0.3329	-0.8914	1.2960
F	1.1369	-0.0465	-1.0285
F	-1.2154	0.9275	-0.4976
F	-0.7950	-1.2532	-0.5846

Transition states (PPs = atoms 3 and 5):

F = 0 pN
EFEI energy (au): -840.66919449
Coordinates (Å):

P	-0.0122	-0.0035	0.0184
F	0.3900	1.2368	0.9288
F	0.5315	-0.9374	1.1851
F	1.1385	-0.0511	-1.0218
F	-1.0753	0.9518	-0.6789
F	-0.9334	-1.2224	-0.4228

F = 300 pN
EFEI energy (au): -840.69054100
Coordinates (Å):

P	-0.0178	0.0007	0.0161
F	0.4050	1.2445	0.9122
F	0.5101	-0.9322	1.1966
F	1.1331	-0.0468	-1.0242
F	-1.0962	0.9564	-0.6668
F	-0.9230	-1.2231	-0.4440

F = 600 pN
EFEI energy (au): -840.71186929
Coordinates (Å):

P	-0.0186	0.0008	0.0168

F	0.4284	1.2483	0.8959
F	0.4887	-0.9299	1.2134
F	1.1325	-0.0468	-1.0237
F	-1.1152	0.9558	-0.6471
F	-0.9046	-1.2287	-0.4655

F = 900 pN
EFEI energy (au): -840.73314384
Coordinates (Å):

P	-0.0193	0.0008	0.0174
F	0.4590	1.2528	0.8740
F	0.4584	-0.9251	1.2344
F	1.1324	-0.0468	-1.0236
F	-1.1392	0.9532	-0.6189
F	-0.8800	-1.2354	-0.4935

F = 1200 pN
EFEI energy (au): -840.75426507
Coordinates (Å):

P	-0.0187	0.0008	0.0169
F	0.5168	1.2593	0.8308
F	0.3924	-0.9095	1.2706
F	1.1348	-0.0469	-1.0258
F	-1.1825	0.9422	-0.5564
F	-0.8316	-1.2463	-0.5463

Reaction U3:

Reactants (PPs = atoms 10 and 18):

F = 0 pN
EFEI energy (au): -661.78347802
Coordinates (Å):

C	3.0340	-0.7181	0.3022
C	2.9439	-2.0272	-0.1737
C	1.7033	-2.5426	-0.5447
C	0.5771	-1.7384	-0.4303
C	0.6210	-0.4197	0.0439
C	1.8897	0.0691	0.4051
H	3.9953	-0.3086	0.5956
H	3.8319	-2.6461	-0.2571
H	1.5889	-3.5517	-0.9252
F	-0.6100	-2.2698	-0.8080
C	-0.6060	0.4069	0.1710
H	1.9687	1.0845	0.7742
C	-0.6201	1.7657	-0.1746

C	-1.7456	2.5696	-0.0508
C	-2.9254	2.0121	0.4389
C	-2.9568	0.6624	0.7933
C	-1.8148	-0.1233	0.6576
F	0.5058	2.3395	-0.6616
H	-1.6788	3.6117	-0.3436
H	-3.8119	2.6302	0.5417
H	-3.8701	0.2198	1.1778
H	-1.8484	-1.1703	0.9335

F = 500 pN
EFEI energy (au): -661.83988421
Coordinates (Å):

C	3.0497	-0.6918	0.1709
C	2.9587	-2.0401	-0.1726
C	1.7057	-2.5964	-0.4142
C	0.5683	-1.8049	-0.3156
C	0.6108	-0.4340	0.0171
C	1.8994	0.0863	0.2618
H	4.0163	-0.2413	0.3732
H	3.8492	-2.6563	-0.2482
H	1.5820	-3.6402	-0.6813
F	-0.6092	-2.4430	-0.5604
C	-0.5997	0.4237	0.1444
H	1.9901	1.1285	0.5348
C	-0.5948	1.8208	-0.0564
C	-1.7292	2.6104	0.0831
C	-2.9397	2.0249	0.4432
C	-2.9911	0.6490	0.6632
C	-1.8449	-0.1265	0.5157
F	0.5411	2.4875	-0.4013
H	-1.6368	3.6761	-0.0961
H	-3.8275	2.6395	0.5537
H	-3.9228	0.1745	0.9544
H	-1.9038	-1.1910	0.6958

F = 1000 pN
EFEI energy (au): -661.89942119
Coordinates (Å):

C	3.0596	-0.6768	0.0325
C	2.9719	-2.0506	-0.1808
C	1.7135	-2.6333	-0.2863
C	0.5652	-1.8564	-0.1862
C	0.6020	-0.4506	0.0181
C	1.9035	0.0905	0.1282
H	4.0275	-0.1949	0.1285

H	3.8640	-2.6637	-0.2596
H	1.5873	-3.6985	-0.4471
F	-0.5926	-2.5801	-0.2990
C	-0.5907	0.4404	0.1459
H	2.0009	1.1520	0.3004
C	-0.5732	1.8596	0.0759
C	-1.7188	2.6347	0.2138
C	-2.9539	2.0359	0.4376
C	-3.0204	0.6473	0.5265
C	-1.8676	-0.1178	0.3842
F	0.5623	2.5987	-0.1274
H	-1.6091	3.7113	0.1412
H	-3.8441	2.6477	0.5446
H	-3.9685	0.1518	0.7099
H	-1.9471	-1.1915	0.4643

Transition states (PPs = atoms 10 and 18):

F = 0 pN
EFEI energy (au): -661.77807806
Coordinates (Å):

C	3.0631	-0.6568	-0.2490
C	2.9737	-2.0456	-0.3108
C	1.7156	-2.6333	-0.2505
C	0.5840	-1.8368	-0.1313
C	0.6117	-0.4261	-0.0642
C	1.9161	0.1220	-0.1296
H	4.0303	-0.1665	-0.2933
H	3.8620	-2.6623	-0.4041
H	1.5765	-3.7080	-0.2932
F	-0.5848	-2.5283	-0.0820
C	-0.6116	0.4261	0.0642
H	2.0356	1.1909	-0.0861
C	-0.5839	1.8367	0.1313
C	-1.7156	2.6333	0.2500
C	-2.9738	2.0456	0.3099
C	-3.0631	0.6568	0.2487
C	-1.9161	-0.1220	0.1299
F	0.5849	2.5282	0.0831
H	-1.5765	3.7080	0.2925
H	-3.8621	2.6623	0.4025
H	-4.0304	0.1666	0.2930
H	-2.0357	-1.1910	0.0863

F = 500 pN
EFEI energy (au): -661.83814197

Coordinates (Å):

C	3.0570	-0.6577	-0.2485
C	2.9715	-2.0465	-0.3104
C	1.7152	-2.6386	-0.2505
C	0.5750	-1.8533	-0.1314
C	0.6034	-0.4375	-0.0642
C	1.9056	0.1137	-0.1293
H	4.0224	-0.1635	-0.2927
H	3.8613	-2.6610	-0.4034
H	1.5832	-3.7142	-0.2938
F	-0.5827	-2.5748	-0.0848
C	-0.6034	0.4375	0.0640
H	2.0144	1.1849	-0.0846
C	-0.5751	1.8533	0.1312
C	-1.7152	2.6386	0.2502
C	-2.9715	2.0465	0.3101
C	-3.0571	0.6577	0.2483
C	-1.9056	-0.1137	0.1291
F	0.5827	2.5748	0.0846
H	-1.5833	3.7142	0.2935
H	-3.8613	2.6610	0.4031
H	-4.0225	0.1635	0.2924
H	-2.0144	-1.1848	0.0844

F = 1000 pN
EFEI energy (au): -661.89924360
Coordinates (Å):

C	3.0517	-0.6591	-0.2483
C	2.9697	-2.0478	-0.3103
C	1.7154	-2.6441	-0.2507
C	0.5669	-1.8700	-0.1315
C	0.5955	-0.4486	-0.0642
C	1.8961	0.1053	-0.1292
H	4.0151	-0.1610	-0.2922
H	3.8610	-2.6602	-0.4033
H	1.5904	-3.7206	-0.2944
F	-0.5792	-2.6219	-0.0871
C	-0.5955	0.4487	0.0640
H	1.9954	1.1784	-0.0838
C	-0.5669	1.8700	0.1316
C	-1.7154	2.6441	0.2506
C	-2.9698	2.0479	0.3101
C	-3.0517	0.6591	0.2479
C	-1.8961	-0.1053	0.1287
F	0.5792	2.6219	0.0874
H	-1.5904	3.7206	0.2945

H	-3.8611	2.6602	0.4031
H	-4.0151	0.1610	0.2915
H	-1.9954	-1.1784	0.0832

<u>Reaction U4</u>:

Reactants (PPs = atoms 12 and 18):

F = 0 pN
EFEI energy (au): -661.78351658
Coordinates (Å):

C	2.6892	-1.2485	1.3042
C	2.9447	-2.0384	0.1822
C	2.0486	-2.0396	-0.8864
C	0.9114	-1.2456	-0.8142
C	0.6249	-0.4309	0.2881
C	1.5426	-0.4570	1.3505
H	3.3844	-1.2412	2.1377
H	3.8374	-2.6545	0.1358
H	2.2079	-2.6490	-1.7693
F	0.0396	-1.2877	-1.8426
C	-0.6061	0.3944	0.3587
H	1.3518	0.1727	2.2143
C	-0.9729	1.2715	-0.6695
C	-2.1098	2.0669	-0.6088
C	-2.9225	2.0021	0.5227
C	-2.5855	1.1480	1.5739
C	-1.4410	0.3568	1.4870
F	-0.1815	1.3745	-1.7569
H	-2.3334	2.7267	-1.4399
H	-3.8141	2.6187	0.5801
H	-3.2157	1.0909	2.4557
H	-1.1876	-0.3225	2.2954

F = 800 pN
EFEI energy (au): -661.86891741
Coordinates (Å):

C	2.6203	-1.3716	1.3685
C	2.9562	-2.0620	0.2031
C	2.1328	-1.9587	-0.9139
C	1.0024	-1.1530	-0.8500
C	0.6515	-0.3984	0.2805
C	1.4886	-0.5588	1.4129
H	3.2442	-1.4612	2.2523
H	3.8426	-2.6876	0.1677
H	2.3316	-2.5032	-1.8305

F	0.1908	-1.1466	-1.9268
C	-0.5788	0.4420	0.3061
H	1.2819	-0.0685	2.3685
C	-1.0639	1.2306	-0.7608
C	-2.2214	1.9956	-0.6499
C	-2.9434	2.0074	0.5397
C	-2.4985	1.2419	1.6176
C	-1.3429	0.4782	1.4898
F	-0.4310	1.3093	-1.9567
H	-2.5297	2.5782	-1.5112
H	-3.8455	2.6061	0.6192
H	-3.0539	1.2281	2.5500
H	-1.0215	-0.1391	2.3223

F = 1600 pN
EFEI energy (au): -661.96106548
Coordinates (Å):

C	2.5716	-1.4649	1.4062
C	2.9712	-2.0808	0.2197
C	2.2041	-1.8999	-0.9235
C	1.0757	-1.0904	-0.8609
C	0.6630	-0.3912	0.2865
C	1.4452	-0.6439	1.4594
H	3.1446	-1.6229	2.3148
H	3.8557	-2.7092	0.1915
H	2.4390	-2.3861	-1.8640
F	0.3225	-1.0277	-1.9771
C	-0.5702	0.4648	0.2594
H	1.2436	-0.2666	2.4773
C	-1.1425	1.1905	-0.8289
C	-2.3052	1.9438	-0.6697
C	-2.9578	2.0212	0.5542
C	-2.4368	1.3205	1.6402
C	-1.2812	0.5675	1.4767
F	-0.6528	1.2512	-2.1024
H	-2.6750	2.4701	-1.5430
H	-3.8615	2.6145	0.6521
H	-2.9305	1.3467	2.6064
H	-0.9102	0.0014	2.3235

F = 2400 pN
EFEI energy (au): -662.05897116
Coordinates (Å):

C	2.5168	-1.5675	1.4366
C	2.9873	-2.0993	0.2357
C	2.2867	-1.8288	-0.9288

C	1.1562	-1.0216	-0.8659
C	0.6597	-0.4064	0.2970
C	1.3880	-0.7489	1.5024
H	3.0395	-1.7941	2.3611
H	3.8729	-2.7263	0.2140
H	2.5736	-2.2362	-1.8919
F	0.4883	-0.8621	-2.0251
C	-0.5796	0.4639	0.2148
H	1.1924	-0.4826	2.5695
C	-1.2384	1.1218	-0.8916
C	-2.3928	1.8804	-0.6840
C	-2.9670	2.0425	0.5686
C	-2.3692	1.4163	1.6582
C	-1.2236	0.6590	1.4606
F	-0.9024	1.1445	-2.2300
H	-2.8254	2.3460	-1.5629
H	-3.8651	2.6411	0.6838
H	-2.7906	1.5047	2.6545
H	-0.7949	0.1619	2.3218

Transition states (PPs = atoms 12 and 18):

F = 0 pN
EFEI energy (au): -661.76808233
Coordinates (Å):

C	2.2051	-1.5316	-1.8746
C	2.9381	-2.0406	-0.8039
C	2.5123	-1.7445	0.4822
C	1.3806	-0.9583	0.6944
C	0.5990	-0.4157	-0.3497
C	1.0808	-0.7507	-1.6401
H	2.5031	-1.7389	-2.8974
H	3.8196	-2.6529	-0.9650
H	3.0354	-2.1078	1.3600
F	1.0941	-0.7588	1.9931
C	-0.6362	0.4420	-0.2317
H	0.5599	-0.3891	-2.5143
C	-1.2744	0.8852	0.9480
C	-2.4198	1.6801	0.9534
C	-3.0056	2.0864	-0.2362
C	-2.4188	1.6788	-1.4328
C	-1.2780	0.8871	-1.4148
F	-0.8247	0.5733	2.1764
H	-2.8240	1.9609	1.9198
H	-3.8972	2.7052	-0.2279
H	-2.8446	1.9740	-2.3865

H	-0.8755	0.6076	-2.3772

F = 800 pN
EFEI energy (au): -661.86137548
Coordinates (Å):

C	2.2189	-1.5412	-1.8826
C	2.9430	-2.0436	-0.8025
C	2.5064	-1.7402	0.4775
C	1.3724	-0.9528	0.6632
C	0.5975	-0.4151	-0.3856
C	1.0881	-0.7560	-1.6823
H	2.5316	-1.7585	-2.8991
H	3.8257	-2.6565	-0.9540
H	3.0193	-2.0961	1.3642
F	1.0559	-0.7328	1.9515
C	-0.6353	0.4409	-0.2090
H	0.6177	-0.4296	-2.6104
C	-1.2765	0.8864	0.9821
C	-2.4230	1.6825	0.9650
C	-3.0071	2.0877	-0.2252
C	-2.4184	1.6787	-1.4197
C	-1.2787	0.8874	-1.3906
F	-0.8714	0.6055	2.2445
H	-2.8333	1.9675	1.9276
H	-3.8986	2.7066	-0.2175
H	-2.8399	1.9711	-2.3761
H	-0.8651	0.6000	-2.3462

F = 1600 pN
EFEI energy (au): -661.95823475
Coordinates (Å):

C	2.2334	-1.5512	-1.8901
C	2.9474	-2.0467	-0.7997
C	2.4996	-1.7355	0.4738
C	1.3637	-0.9468	0.6327
C	0.5962	-0.4142	-0.4214
C	1.0969	-0.7621	-1.7253
H	2.5617	-1.7794	-2.8996
H	3.8312	-2.6604	-0.9408
H	3.0020	-2.0840	1.3694
F	1.0187	-0.7069	1.9105
C	-0.6344	0.4403	-0.1901
H	0.6778	-0.4714	-2.7029
C	-1.2782	0.8876	1.0140
C	-2.4254	1.6841	0.9775
C	-3.0087	2.0888	-0.2132

C	-2.4196	1.6795	-1.4062
C	-1.2806	0.8887	-1.3692
F	-0.9145	0.6354	2.3081
H	-2.8412	1.9730	1.9366
H	-3.9002	2.7078	-0.2049
H	-2.8384	1.9701	-2.3644
H	-0.8585	0.5954	-2.3200

F = 2400 pN
EFEI energy (au): -662.05852466
Coordinates (Å):

C	2.2488	-1.5620	-1.8972
C	2.9520	-2.0498	-0.7959
C	2.4927	-1.7306	0.4705
C	1.3552	-0.9408	0.6034
C	0.5953	-0.4135	-0.4567
C	1.1074	-0.7696	-1.7684
H	2.5933	-1.8015	-2.8988
H	3.8370	-2.6643	-0.9259
H	2.9843	-2.0717	1.3749
F	0.9842	-0.6829	1.8713
C	-0.6342	0.4402	-0.1748
H	0.7397	-0.5146	-2.7917
C	-1.2801	0.8889	1.0438
C	-2.4275	1.6855	0.9905
C	-3.0109	2.0903	-0.2003
C	-2.4226	1.6817	-1.3927
C	-1.2840	0.8912	-1.3506
F	-0.9546	0.6632	2.3677
H	-2.8483	1.9778	1.9464
H	-3.9024	2.7093	-0.1903
H	-2.8399	1.9714	-2.3519
H	-0.8559	0.5939	-2.2983

Reaction B1:

Reactants (PPs = atoms 1 and 2):

F = 0 pN
EFEI energy (au): -960.40552053
Coordinates (Å):

C	0.5221	0.0063	-0.0006
Cl	2.3790	0.0117	0.0001
H	0.1881	-0.6654	0.7832
H	0.1880	-0.3379	-0.9737
H	0.1838	1.0197	0.1879

Cl	-2.6718	-0.0122	0.0024

F = 500 pN
EFEI energy (au): -960.42698233
Coordinates (Å):

C	0.5344	0.0056	-0.0022
Cl	2.4218	0.0096	0.0011
H	0.2108	-0.6677	0.7834
H	0.2139	-0.3396	-0.9786
H	0.2091	1.0224	0.1867
Cl	-2.6215	-0.0145	0.0038

F = 1000 pN
EFEI energy (au): -960.44884277
Coordinates (Å):

C	0.5110	0.0043	-0.0010
Cl	2.4382	0.0129	-0.0020
H	0.2079	-0.6725	0.7885
H	0.2055	-0.3430	-0.9805
H	0.2001	1.0243	0.1895
Cl	-2.5943	-0.0103	-0.0002

F = 1500 pN
EFEI energy (au): -960.47127837
Coordinates (Å):

C	0.4710	0.0041	-0.0010
Cl	2.4639	0.0129	-0.0018
H	0.1966	-0.6768	0.7935
H	0.1943	-0.3453	-0.9866
H	0.1888	1.0306	0.1907
Cl	-2.5461	-0.0096	-0.0005

Transition states (PPs = atoms 1 and 2):

F = 0 pN
EFEI energy (au): -960.39177544
Coordinates (Å):

C	-0.0000	0.0000	0.0001
Cl	2.3721	0.0083	-0.0006
H	0.0026	-0.6981	0.8162
H	0.0010	-0.3576	-1.0126
H	-0.0037	1.0558	0.1967
Cl	-2.3721	-0.0084	0.0004

F = 500 pN
EFEI energy (au): -960.41874035

Coordinates (Å):

C	0.0509	0.0001	-0.0000
Cl	2.3802	0.0084	-0.0004
H	-0.0004	-0.6977	0.8154
H	-0.0019	-0.3574	-1.0120
H	-0.0067	1.0550	0.1964
Cl	-2.4221	-0.0085	0.0007

F = 1000 pN
EFEI energy (au): -960.44513080
Coordinates (Å):

C	0.1090	0.0004	-0.0000
Cl	2.3795	0.0084	-0.0005
H	0.0001	-0.6955	0.8130
H	-0.0016	-0.3563	-1.0088
H	-0.0062	1.0518	0.1959
Cl	-2.4809	-0.0088	0.0005

F = 1500 pN
EFEI energy (au): -960.47069895
Coordinates (Å):

C	0.2089	0.0007	-0.0000
Cl	2.3880	0.0085	-0.0005
H	0.0325	-0.6907	0.8075
H	0.0308	-0.3538	-1.0020
H	0.0260	1.0448	0.1946
Cl	-2.5359	-0.0088	0.0005

Reaction B2:

Reactants (PPs = atoms 1 and 2):

F = 0 pN
EFEI energy (au): -556.68510359
Coordinates (Å):

C	0.1500	0.0034	-0.0006
Cl	1.9625	0.0095	-0.0006
N	-3.2557	-0.0184	0.0015
H	-0.1992	0.7768	-0.6813
H	-0.1973	0.2045	1.0105
H	-0.1935	-0.9744	-0.3310
H	-3.6309	0.8755	0.3079
H	-3.6244	-0.7323	0.6245
H	-3.6289	-0.2027	-0.9262

F = 1000 pN

EFEI energy (au): -556.72713343
Coordinates (Å):

C	0.1207	0.0064	0.0005
Cl	1.9744	0.0001	-0.0038
N	-3.2403	-0.0210	0.0005
H	-0.2097	0.7849	-0.6821
H	-0.2078	0.2103	1.0162
H	-0.2156	-0.9726	-0.3303
H	-3.6150	0.8732	0.3070
H	-3.6096	-0.7344	0.6237
H	-3.6143	-0.2050	-0.9269

F = 2000 pN
EFEI energy (au): -556.77024052
Coordinates (Å):

C	0.0796	0.0021	-0.0006
Cl	1.9875	0.0112	-0.0008
N	-3.2168	-0.0166	0.0017
H	-0.2363	0.7817	-0.6868
H	-0.2335	0.2043	1.0190
H	-0.2279	-0.9842	-0.3340
H	-3.5951	0.8763	0.3076
H	-3.5849	-0.7310	0.6246
H	-3.5900	-0.2019	-0.9259

F = 3000 pN
EFEI energy (au): -556.81485158
Coordinates (Å):

C	0.0125	0.0003	-0.0010
Cl	2.0020	0.0132	-0.0005
N	-3.1750	-0.0158	0.0018
H	-0.2750	0.7847	-0.6917
H	-0.2717	0.2036	1.0252
H	-0.2635	-0.9927	-0.3365
H	-3.5515	0.8779	0.3079
H	-3.5452	-0.7291	0.6249
H	-3.5498	-0.2001	-0.9253

Transition states (PPs = atoms 1 and 2):

F = 0 pN
EFEI energy (au): -556.63317995
Coordinates (Å):

C	-0.7089	-0.0028	-0.0000
Cl	1.8584	0.0074	0.0002
H	-0.4966	-0.6901	0.8054

H	-0.4977	-0.3554	-0.9986
H	-0.5034	1.0396	0.1932
N	-2.4765	-0.0099	-0.0002
H	-2.8378	0.6092	-0.7266
H	-2.8369	0.3073	0.9002
H	-2.8317	-0.9504	-0.1746

F = 1000 pN
EFEI energy (au): -556.69187168
Coordinates (Å):

C	-0.6074	-0.0023	-0.0001
Cl	1.9507	0.0076	0.0004
H	-0.4753	-0.6968	0.8140
H	-0.4765	-0.3593	-1.0090
H	-0.4823	1.0507	0.1947
N	-2.5447	-0.0101	-0.0003
H	-2.9010	0.6092	-0.7261
H	-2.8999	0.3066	0.9002
H	-2.8947	-0.9505	-0.1750

F = 2000 pN
EFEI energy (au): -556.75058483
Coordinates (Å):

C	-0.5327	-0.0021	-0.0001
Cl	2.0247	0.0082	0.0002
H	-0.4857	-0.7010	0.8185
H	-0.4869	-0.3612	-1.0148
H	-0.4926	1.0566	0.1961
N	-2.6723	-0.0106	-0.0004
H	-3.0288	0.6077	-0.7254
H	-3.0278	0.3060	0.8987
H	-3.0227	-0.9496	-0.1746

F = 3000 pN
EFEI energy (au): -556.80861805
Coordinates (Å):

C	-0.4114	-0.0017	-0.0001
Cl	2.0695	0.0083	0.0003
H	-0.4727	-0.7011	0.8178
H	-0.4736	-0.3607	-1.0145
H	-0.4796	1.0559	0.1965
N	-2.8408	-0.0113	-0.0003
H	-3.2056	0.6042	-0.7230
H	-3.2045	0.3046	0.8952
H	-3.1995	-0.9470	-0.1734

<u>Reaction B3</u>:

Reactants (PPs = atoms 8 and 9):

F = 0 pN
EFEI energy (au): -573.43160767
Coordinates (Å):

C	2.2457	-1.1847	0.2493
C	1.5150	-0.7306	1.2945
C	1.5225	0.7378	1.2897
C	2.2579	1.1775	0.2416
C	2.8005	-0.0089	-0.5119
H	3.9012	-0.0146	-0.5185
H	2.4965	-0.0107	-1.5695
C	-1.0349	0.6716	-1.4658
C	-1.0350	-0.6661	-1.4694
C	-1.6852	-1.1331	-0.2140
O	-1.9056	-2.2479	0.1711
O	-2.0539	-0.0026	0.5155
C	-1.6851	1.1318	-0.2078
O	-1.9051	2.2445	0.1836
H	-0.6567	-1.3578	-2.2098
H	-0.6566	1.3673	-2.2023
H	2.4565	2.2094	-0.0231
H	1.0140	1.3574	2.0201
H	1.0003	-1.3402	2.0290
H	2.4338	-2.2203	-0.0085

F = 3000 pN
EFEI energy (au): -573.52484934
Coordinates (Å):

C	2.2854	-1.1865	0.2434
C	1.5664	-0.7360	1.2986
C	1.5731	0.7318	1.2984
C	2.2964	1.1754	0.2432
C	2.8326	-0.0081	-0.5192
H	3.9332	-0.0135	-0.5342
H	2.5205	-0.0064	-1.5743
C	-0.9347	0.6869	-1.4568
C	-0.9361	-0.6867	-1.4556
C	-1.6077	-1.1337	-0.2082
O	-1.8405	-2.2445	0.1836
O	-1.9840	0.0027	0.5083
C	-1.6054	1.1373	-0.2101
O	-1.8357	2.2491	0.1800
H	-0.5476	-1.3792	-2.1893

H	-0.5446	1.3774	-2.1914
H	2.4916	2.2083	-0.0200
H	1.0722	1.3489	2.0360
H	1.0600	-1.3483	2.0365
H	2.4713	-2.2212	-0.0194

F = 6000 pN
EFEI energy (au): -573.62086959
Coordinates (Å):

C	2.2567	-1.1860	0.2336
C	1.5528	-0.7370	1.3000
C	1.5571	0.7301	1.3004
C	2.2638	1.1755	0.2342
C	2.7922	-0.0067	-0.5355
H	3.8926	-0.0101	-0.5640
H	2.4669	-0.0052	-1.5864
C	-0.8888	0.7098	-1.4431
C	-0.8918	-0.7096	-1.4406
C	-1.5889	-1.1358	-0.2050
O	-1.8376	-2.2426	0.1910
O	-1.9734	0.0057	0.4995
C	-1.5839	1.1432	-0.2089
O	-1.8274	2.2524	0.1836
H	-0.4921	-1.4009	-2.1686
H	-0.4863	1.3968	-2.1736
H	2.4535	2.2089	-0.0310
H	1.0655	1.3462	2.0450
H	1.0577	-1.3504	2.0445
H	2.4404	-2.2204	-0.0319

F = 9000 pN
EFEI energy (au): -573.72061579
Coordinates (Å):

C	2.2122	-1.1857	0.2180
C	1.5276	-0.7369	1.2980
C	1.5325	0.7286	1.2994
C	2.2203	1.1749	0.2205
C	2.7386	-0.0064	-0.5569
H	3.8388	-0.0103	-0.5961
H	2.4028	-0.0036	-1.6041
C	-0.8287	0.7418	-1.4218
C	-0.8319	-0.7427	-1.4181
C	-1.5611	-1.1412	-0.1988
O	-1.8301	-2.2428	0.2027
O	-1.9564	0.0066	0.4901
C	-1.5559	1.1494	-0.2043

O	-1.8199	2.2540	0.1920
H	-0.4188	-1.4320	-2.1395
H	-0.4127	1.4258	-2.1467
H	2.4061	2.2086	-0.0461
H	1.0532	1.3444	2.0521
H	1.0444	-1.3510	2.0496
H	2.3913	-2.2201	-0.0504

F = 12000 pN
EFEI energy (au): -573.82652434
Coordinates (Å):

C	2.1152	-1.1857	0.1835
C	1.4696	-0.7336	1.2891
C	1.4756	0.7268	1.2879
C	2.1249	1.1717	0.1816
C	2.6314	-0.0097	-0.6018
H	3.7317	-0.0142	-0.6432
H	2.2922	-0.0092	-1.6466
C	-0.7084	0.8129	-1.3711
C	-0.7133	-0.8072	-1.3720
C	-1.5067	-1.1530	-0.1909
O	-1.8220	-2.2444	0.2143
O	-1.9159	0.0054	0.4739
C	-1.4996	1.1621	-0.1895
O	-1.8083	2.2549	0.2171
H	-0.2868	-1.4921	-2.0883
H	-0.2774	1.4961	-2.0864
H	2.3034	2.2054	-0.0897
H	1.0194	1.3451	2.0526
H	1.0081	-1.3469	2.0547
H	2.2850	-2.2213	-0.0862

Transition states (PPs = atoms 8 and 9):

F = 0 pN
EFEI energy (au): -573.40713246
Coordinates (Å):

C	1.6189	-1.1589	-0.0246
C	1.3103	-0.7005	1.2688
C	1.3125	0.7003	1.2667
C	1.6224	1.1538	-0.0282
C	2.2715	-0.0047	-0.7503
H	3.3447	-0.0059	-0.5000
H	2.1831	-0.0062	-1.8378
C	-0.2635	0.7004	-1.0931
C	-0.2657	-0.7029	-1.0911

C	-1.2885	-1.1403	-0.1164
O	-1.6929	-2.2397	0.1583
O	-1.7802	0.0035	0.5259
C	-1.2849	1.1438	-0.1197
O	-1.6858	2.2453	0.1519
H	-0.0137	-1.3482	-1.9218
H	-0.0095	1.3425	-1.9257
H	1.7918	2.1941	-0.2826
H	0.9972	1.3301	2.0903
H	0.9931	-1.3268	2.0944
H	1.7851	-2.2005	-0.2758

F = 3000 pN
EFEI energy (au): 573.50508873
Coordinates (Å):

C	1.6456	-1.1616	-0.0125
C	1.3151	-0.7028	1.2695
C	1.3172	0.7026	1.2674
C	1.6492	1.1566	-0.0159
C	2.2804	-0.0046	-0.7467
H	3.3583	-0.0060	-0.5146
H	2.1779	-0.0061	-1.8329
C	-0.2843	0.7214	-1.1123
C	-0.2865	-0.7239	-1.1101
C	-1.2962	-1.1439	-0.1205
O	-1.7036	-2.2401	0.1656
O	-1.7747	0.0035	0.5259
C	-1.2927	1.1475	-0.1240
O	-1.6968	2.2458	0.1588
H	-0.0182	-1.3728	-1.9318
H	-0.0140	1.3671	-1.9360
H	1.8164	2.1963	-0.2730
H	0.9941	1.3311	2.0890
H	0.9901	-1.3278	2.0930
H	1.8096	-2.2026	-0.2664

F = 6000 pN
EFEI energy (au): -573.6063218
Coordinates (Å):

C	1.6819	-1.1649	0.0039
C	1.3224	-0.7056	1.2709
C	1.3245	0.7056	1.2687
C	1.6852	1.1598	0.0001
C	2.2942	-0.0046	-0.7412
H	3.3779	-0.0058	-0.5344
H	2.1713	-0.0062	-1.8253

C	-0.3119	0.7487	-1.1368
C	-0.3142	-0.7510	-1.1347
C	-1.3075	-1.1485	-0.1262
O	-1.7175	-2.2408	0.1750
O	-1.7705	0.0034	0.5237
C	-1.3042	1.1520	-0.1296
O	-1.7112	2.2464	0.1683
H	-0.0256	-1.4047	-1.9446
H	-0.0216	1.3991	-1.9487
H	1.8494	2.1988	-0.2605
H	0.9913	1.3326	2.0873
H	0.9875	-1.3290	2.0916
H	1.8431	-2.2052	-0.2534

F = 9000 pN
EFEI energy (au): -573.7120586
Coordinates (Å):

C	1.7397	-1.1694	0.0289
C	1.3355	-0.7107	1.2730
C	1.3371	0.7092	1.2709
C	1.7419	1.1634	0.0252
C	2.3217	-0.0048	-0.7299
H	3.4127	-0.0054	-0.5623
H	2.1669	-0.0065	-1.8100
C	-0.3558	0.7889	-1.1732
C	-0.3592	-0.7911	-1.1713
C	-1.3275	-1.1549	-0.1353
O	-1.7391	-2.2413	0.1897
O	-1.7708	0.0040	0.5161
C	-1.3235	1.1592	-0.1387
O	-1.7315	2.2481	0.1827
H	-0.0429	-1.4522	-1.9635
H	-0.0382	1.4464	-1.9680
H	1.9030	2.2014	-0.2396
H	0.9882	1.3340	2.0846
H	0.9850	-1.3323	2.0885
H	1.8974	-2.2086	-0.2335

F = 12000 pN
EFEI energy (au): -573.82552537
Coordinates (Å):

C	1.9206	-1.1766	0.0999
C	1.3849	-0.7221	1.2741
C	1.3867	0.7206	1.2727
C	1.9234	1.1715	0.0977
C	2.4448	-0.0039	-0.6836

H	3.5471	-0.0052	-0.6422
H	2.1799	-0.0046	-1.7447
C	-0.5097	0.8701	-1.2751
C	-0.5124	-0.8716	-1.2734
C	-1.4002	-1.1678	-0.1605
O	-1.7880	-2.2452	0.2282
O	-1.8117	0.0030	0.4861
C	-1.3965	1.1712	-0.1627
O	-1.7809	2.2509	0.2240
H	-0.1305	-1.5514	-2.0183
H	-0.1256	1.5474	-2.0213
H	2.0839	2.2076	-0.1752
H	0.9837	1.3406	2.0650
H	0.9805	-1.3396	2.0676
H	2.0787	-2.2135	-0.1709

Reaction B4:

Reactants (PPs = atoms 1 and 3):

F = 0 pN
EFEI energy (au): -287.73518088
Coordinates (Å):

C	-2.3507	0.5158	2.0482
H	-2.7415	0.8036	3.0213
C	-1.8332	-0.6948	1.8238
H	-1.7846	-1.4409	2.6140
H	-1.4423	-0.9817	0.8502
H	-2.4115	1.2632	1.2607
O	0.1291	-0.9244	-1.1118
C	1.1476	0.9141	-2.0211
H	0.5477	0.8164	-2.9151
H	1.8813	1.6954	-1.8895
N	0.9646	0.0403	-1.0686
C	1.7542	0.1197	0.1852
H	2.4475	0.9609	0.1568
H	2.2909	-0.8244	0.2883
H	1.0451	0.2247	1.0076

F = 3000 pN
EFEI energy (au): -287.82823234
Coordinates (Å):

C	-2.3490	0.5225	2.0403
H	-2.7377	0.8066	3.0144
C	-1.8205	-0.7210	1.8126
H	-1.7768	-1.4606	2.6081

H	-1.4311	-1.0043	0.8378
H	-2.4054	1.2636	1.2476
O	0.1315	-0.9401	-1.1168
C	1.1433	0.9042	-2.0216
H	0.5438	0.8063	-2.9157
H	1.8744	1.6876	-1.8884
N	0.9637	0.0276	-1.0712
C	1.7527	0.1067	0.1830
H	2.4428	0.9507	0.1568
H	2.2930	-0.8355	0.2838
H	1.0430	0.2071	1.0055

F = 6000 pN
EFEI energy (au): -287.92393328
Coordinates (Å):

C	-2.3483	0.5453	2.0383
H	-2.7351	0.8235	3.0140
C	-1.8096	-0.7401	1.8042
H	-1.7749	-1.4736	2.6046
H	-1.4219	-1.0176	0.8280
H	-2.3966	1.2808	1.2411
O	0.1327	-0.9441	-1.1156
C	1.1373	0.9055	-2.0178
H	0.5381	0.8065	-2.9122
H	1.8653	1.6917	-1.8834
N	0.9609	0.0267	-1.0685
C	1.7498	0.1079	0.1858
H	2.4365	0.9546	0.1600
H	2.2939	-0.8323	0.2855
H	1.0397	0.2047	1.0083

F = 9000 pN
EFEI energy (au): -288.02308171
Coordinates (Å):

C	-2.3481	0.5746	2.0358
H	-2.7328	0.8442	3.0136
C	-1.7947	-0.7677	1.7919
H	-1.7710	-1.4936	2.5982
H	-1.4082	-1.0367	0.8136
H	-2.3866	1.3032	1.2332
O	0.1331	-0.9498	-1.1111
C	1.1284	0.9052	-2.0120
H	0.5286	0.8044	-2.9058
H	1.8532	1.6943	-1.8778
N	0.9573	0.0250	-1.0635
C	1.7468	0.1079	0.1901

H	2.4303	0.9572	0.1644
H	2.2946	-0.8303	0.2885
H	1.0370	0.2014	1.0133

F = 12000 pN
EFEI energy (au): -288.12738043
Coordinates (Å):

C	-2.3468	0.6253	2.0352
H	-2.7292	0.8782	3.0166
C	-1.7728	-0.8131	1.7709
H	-1.7702	-1.5293	2.5844
H	-1.3872	-1.0660	0.7889
H	-2.3666	1.3453	1.2262
O	0.1366	-0.9593	-1.1086
C	1.1169	0.9046	-2.0072
H	0.5188	0.7991	-2.9015
H	1.8352	1.6996	-1.8722
N	0.9525	0.0225	-1.0591
C	1.7397	0.1118	0.1955
H	2.4158	0.9670	0.1707
H	2.2954	-0.8217	0.2941
H	1.0279	0.1990	1.0178

Transition states (PPs = atoms 1 and 3):

F = 0 pN
EFEI energy (au): -287.70952469
Coordinates (Å):

C	-1.4289	0.7400	0.7383
H	-0.9397	1.3289	1.5107
C	-1.4515	-0.6430	0.8472
H	-0.9266	-1.1548	1.6455
H	-2.1703	-1.2379	0.2967
H	-2.2379	1.2491	0.2214
O	0.1117	-1.2073	-0.5703
C	-0.0223	1.0127	-0.8551
H	-0.7783	0.8840	-1.6180
H	0.4550	1.9847	-0.7740
N	0.7359	-0.0667	-0.6089
C	1.9014	0.0374	0.2733
H	2.4154	0.9872	0.1108
H	2.5715	-0.7929	0.0491
H	1.5703	-0.0413	1.3168

F = 3000 pN
EFEI energy (au): -287.80628971

Coordinates (Å):

C	-1.4569	0.7606	0.7560
H	-0.9637	1.3479	1.5256
C	-1.4703	-0.6615	0.8643
H	-0.9328	-1.1662	1.6579
H	-2.1785	-1.2556	0.3006
H	-2.2580	1.2605	0.2204
O	0.1237	-1.2066	-0.5770
C	-0.0010	1.0175	-0.8720
H	-0.7713	0.8860	-1.6188
H	0.4801	1.9867	-0.7910
N	0.7378	-0.0639	-0.6024
C	1.9109	0.0372	0.2708
H	2.4175	0.9923	0.1170
H	2.5836	-0.7868	0.0312
H	1.5858	-0.0570	1.3143

F = 6000 pN
EFEI energy (au): -287.90605163
Coordinates (Å):

C	-1.4937	0.7844	0.7769
H	-0.9960	1.3687	1.5442
C	-1.4951	-0.6874	0.8826
H	-0.9443	-1.1838	1.6712
H	-2.1893	-1.2792	0.3008
H	-2.2844	1.2736	0.2185
O	0.1384	-1.2079	-0.5863
C	0.0255	1.0205	-0.8963
H	-0.7633	0.8866	-1.6219
H	0.5113	1.9864	-0.8154
N	0.7405	-0.0624	-0.5976
C	1.9233	0.0356	0.2641
H	2.4212	0.9964	0.1200
H	2.5982	-0.7810	0.0063
H	1.6064	-0.0764	1.3079

F = 9000 pN
EFEI energy (au): -288.00983463
Coordinates (Å):

C	-1.5461	0.8207	0.8107
H	-1.0424	1.3983	1.5773
C	-1.5284	-0.7219	0.9082
H	-0.9623	-1.2080	1.6913
H	-2.2029	-1.3085	0.3001
H	-2.3223	1.2962	0.2233
O	0.1580	-1.2084	-0.5925

C	0.0656	1.0239	-0.9320
H	-0.7496	0.8878	-1.6259
H	0.5576	1.9855	-0.8520
N	0.7464	-0.0600	-0.5904
C	1.9416	0.0352	0.2566
H	2.4275	1.0037	0.1252
H	2.6200	-0.7706	-0.0250
H	1.6358	-0.0999	1.3001

F = 12000 pN
EFEI energy (au): -288.12008722
Coordinates (Å):

C	-1.6645	0.8897	0.8847
H	-1.1575	1.4542	1.6562
C	-1.5971	-0.7863	0.9575
H	-1.0051	-1.2528	1.7320
H	-2.2245	-1.3626	0.2936
H	-2.4102	1.3387	0.2426
O	0.1994	-1.2115	-0.6072
C	0.1489	1.0230	-1.0123
H	-0.7154	0.8894	-1.6431
H	0.6508	1.9774	-0.9317
N	0.7653	-0.0580	-0.5890
C	1.9813	0.0339	0.2341
H	2.4458	1.0154	0.1277
H	2.6655	-0.7512	-0.0895
H	1.6949	-0.1436	1.2751

Reaction B5:

1,3 butadiene (not subject to F)
energy (au): -156.00501129
Coordinates (Å):

C	1.5485	-0.4935	-0.0802
C	0.7280	0.5519	0.1047
H	1.1604	1.5035	0.4141
C	-0.7280	0.5519	-0.1047
H	-1.1604	1.5035	-0.4141
C	-1.5485	-0.4935	0.0802
H	-2.6137	-0.4150	-0.1136
H	-1.1831	-1.4506	0.4432
H	2.6137	-0.4149	0.1136
H	1.1831	-1.4506	-0.4432

Ethene (PPs = atoms 1 and 3):

F = 0 pN
EFEI energy (au): -78.59970486
Coordinates (Å):

C	-0.2141	0.6185	0.1343
H	-0.5905	0.8924	1.1164
C	0.2126	-0.6169	-0.1336
H	0.2018	-1.4013	0.6188
H	0.5895	-0.8910	-1.1157
H	-0.2034	1.4025	-0.6184

F = 3000 pN
EFEI energy (au): -78.69264965
Coordinates (Å):

C	-0.2192	0.6343	0.1378
H	-0.5943	0.9019	1.1214
C	0.2179	-0.6328	-0.1372
H	0.2043	-1.4123	0.6192
H	0.5940	-0.9002	-1.1205
H	-0.2068	1.4134	-0.6189

F = 6000 pN
EFEI energy (au): -78.78821727
Coordinates (Å):

C	-0.2256	0.6532	0.1417
H	-0.5994	0.9144	1.1267
C	0.2255	-0.6532	-0.1417
H	0.2095	-1.4277	0.6185
H	0.5998	-0.9142	-1.1266
H	-0.2101	1.4276	-0.6187

F = 9000 pN
EFEI energy (au): -78.88718895
Coordinates (Å):

C	-0.2350	0.6801	0.1475
H	-0.6065	0.9299	1.1352
C	0.2350	-0.6801	-0.1476
H	0.2144	-1.4460	0.6198
H	0.6065	-0.9298	-1.1352
H	-0.2146	1.4460	-0.6199

F = 12000 pN
EFEI energy (au): -78.99119169
Coordinates (Å):

C	-0.2504	0.7246	0.1572
H	-0.6185	0.9581	1.1486

C	0.2504	-0.7246	-0.1572
H	0.2235	-1.4783	0.6201
H	0.6185	-0.9581	-1.1487
H	-0.2235	1.4783	-0.6201

Transition states (PPs = atoms 5 and 6):

F = 0 pN
EFEI energy (au): -234.57085598
BSSE correction (au): 0.000998949011
Coordinates (Å):

C	-1.2236	0.6601	-0.7542
C	-1.2121	0.6768	0.6325
C	-0.0121	0.6766	1.3672
C	1.2282	0.6597	0.7470
C	-0.0677	-1.2247	-1.2216
C	1.1186	-1.2249	-0.4952
H	2.1287	0.5355	1.3425
H	1.3786	1.1648	-0.1999
H	-0.0660	0.4642	2.4334
H	-2.1372	0.4645	1.1651
H	-0.4485	1.1650	-1.3186
H	-2.1634	0.5362	-1.2855
H	-0.0523	-0.9757	-2.2784
H	-0.8954	-1.8490	-0.9055
H	2.0529	-0.9759	-0.9894
H	1.2133	-1.8493	0.3856

F = 3000 pN
EFEI energy (au): -234.66791870
BSSE correction (au): 0.000972194935
Coordinates (Å):

C	-1.2354	0.6784	-0.7500
C	-1.2146	0.6796	0.6331
C	-0.0116	0.6795	1.3697
C	1.2298	0.6781	0.7595
C	-0.0778	-1.2470	-1.2439
C	1.1430	-1.2472	-0.4965
H	2.1291	0.5592	1.3576
H	1.3797	1.1627	-0.1977
H	-0.0686	0.4632	2.4349
H	-2.1375	0.4635	1.1682
H	-0.4508	1.1630	-1.3186
H	-2.1769	0.5597	-1.2790
H	-0.0592	-0.9869	-2.2971
H	-0.9075	-1.8610	-0.9160

H	2.0727	-0.9872	-0.9919
H	1.2281	-1.8613	0.3915

F = 6000 pN
EFEI energy (au): -234.76811362
BSSE correction (au): 0.000932186156
Coordinates (Å):

C	-1.2504	0.7018	-0.7436
C	-1.2183	0.6842	0.6351
C	-0.0116	0.6841	1.3743
C	1.2311	0.7019	0.7767
C	-0.0913	-1.2752	-1.2723
C	1.1742	-1.2754	-0.4985
H	2.1284	0.5898	1.3786
H	1.3809	1.1606	-0.1929
H	-0.0729	0.4645	2.4386
H	-2.1387	0.4647	1.1731
H	-0.4547	1.1607	-1.3173
H	-2.1942	0.5901	-1.2696
H	-0.0690	-1.0020	-2.3213
H	-0.9228	-1.8767	-0.9294
H	2.0978	-1.0017	-0.9959
H	1.2478	-1.8768	0.3981

F = 9000 pN
EFEI energy (au): -234.87257379
BSSE correction (au): 0.000865503192
Coordinates (Å):

C	-1.2720	0.7355	-0.7341
C	-1.2236	0.6917	0.6380
C	-0.0114	0.6916	1.3802
C	1.2327	0.7353	0.7996
C	-0.1087	-1.3161	-1.3159
C	1.2207	-1.3167	-0.5023
H	2.1271	0.6330	1.4071
H	1.3836	1.1586	-0.1855
H	-0.0783	0.4696	2.4438
H	-2.1406	0.4697	1.1810
H	-0.4632	1.1590	-1.3163
H	-2.2197	0.6334	-1.2546
H	-0.0800	-1.0246	-2.3587
H	-0.9430	-1.8997	-0.9526
H	2.1364	-1.0253	-1.0020
H	1.2763	-1.9003	0.4060

F = 12000 pN

EFEI energy (au): -234.98430885
BSSE correction (au): 0.000754722817
Coordinates (Å):

C	-1.3176	0.8080	-0.7091
C	-1.2382	0.7118	0.6489
C	-0.0152	0.7117	1.3982
C	1.2308	0.8075	0.8522
C	-0.1449	-1.4097	-1.4151
C	1.3263	-1.4103	-0.5160
H	2.1170	0.7268	1.4740
H	1.3892	1.1584	-0.1598
H	-0.0947	0.4918	2.4614
H	-2.1494	0.4917	1.2026
H	-0.4880	1.1595	-1.3096
H	-2.2740	0.7277	-1.2164
H	-0.1026	-1.0872	-2.4465
H	-0.9833	-1.9530	-1.0057
H	2.2248	-1.0867	-1.0235
H	1.3441	-1.9531	0.4171
